# A cutback in Imiquimod cutaneous toxicity; comparative cutaneous toxicity analysis of Imiquimod nanotransethosomal gel with 5% marketed cream on the BALB/c mice

**DOI:** 10.1038/s41598-022-18671-1

**Published:** 2022-08-20

**Authors:** Humzah Jamshaid, Fakhar ud Din, Maimoona Malik, Muhammad Mukhtiar, Han Gon Choi, Tofeeq Ur-Rehman, Gul Majid Khan

**Affiliations:** 1 Nanomedicine Research Group, Department of Pharmacy, Quaid-I-Azam University, Islamabad, 45320 Pakistan; 2 Department of Pharmacy, Ibadat International University, Islamabad, Pakistan; 3 Department of Pharmacy, Faculty of Medical and Health Sciences, University of Poonch Rawalakot, Rawalakot, AJK Pakistan; 4 College of Pharmacy, Hanyang University, 55 Hanyangdaehak-ro, Sangnok-gu, Ansan, 15588 South Korea; 5 Islamia College University, Peshawar, Khyber Pakhtunkhwa Pakistan

**Keywords:** Drug discovery, Diseases, Medical research

## Abstract

Herein, Imiquimod (IMQ) was incorporated in nanotransethosomes (nTES) to develop the IMQ-nTES nano-drug delivery system. IMQ-nTES was optimized using 2^3^ factorial design. The optimized formulation was expressed with a particle size of 192.4 ± 1.60 nm, Poly-dispersibility of 0.115 ± 0.008, and IMQ percent entrapment efficiency of 91.05 ± 3.22%. Smooth and round morphology of IMQ-nTES vesicles was confirmed by TEM micrographs. Moreover, FTIR results have shown drug-excipient compatibility. The IMQ-nTES was laden inside the low molecular weight chitosan gel, which exhibited easy application, spreadability and no irritation to the applied skin. The release pattern has clearly exhibited improved dissolution properties of IMQ with the provision of the sustain release pattern. Higher IMQ content was deposited in deeper epidermis and dermis with IMQ-nTES gel, in contrast to ALDARA. In vivo, comparative toxicity study on BALB/c mice has shown significantly reduced (p < 0.001) psoriatic area severity index (PASI) score and less increment in ear thickness. Epidermal hyperplasia was an obvious finding with ALDARA which was, providentially, minimal in IMQ-nTES gel-treated skin. FTIR analysis of skin tissue has shown an enhancement of lipid and protein content in the ALDARA group, however, in the IMQ-nTES group no such change was observed. With ALDARA application, CD4^+^ T-cells and constitutive NF-κβ expression were significantly elevated, in comparison to the IMQ-nTES gel treated group. Moreover, the adequate expression of IFN-γ and cytotoxic CD8^+^ T-cells were suggesting the preserved IMQ efficacy with IMQ-nTES gel. Quantification of cutaneous as well as systemic inflammatory markers has also suggested the reduced psoriatic potential of IMQ-nTES gel. In essence, IMQ-nTES gel can be a suitable alternative to ALDARA owing to its better safety profile.

## Introduction

Imidazoquinolone structural compound named Imiquimod (IMQ) also referred to as S-26308, is an immunomodulatory agent that possesses significant antiviral and antitumor potential. Augmentation of the body’s innate and acquired immune response, particularly the cutaneous immune system, is a major pretext for imiquimod’s antineoplastic and antiviral effect^1^. By 1997, Food and Drug Administration (FDA) approved IMQ for the treatment of human papillomavirus (HPV) induced perianal and pregenital warts (genital warts). Furthermore, this novel immune activator has also proven its effectiveness in pre-cancerous skin disorders which is termed actinic keratosis (AK)^2^, and superficial basal cell carcinoma (BCC)^3^. At present, IMQ has also gained attraction as a charismatic candidate for transcutaneous immunization (TCI) against several viral infections^4^. Commercially, it is available in the form of a 5% topical cream, registered under the brand name ALDARA^5,6^. Macrophages, lymphocytes and dendritic cells expressed toll-like receptor-7 and 8 (TLR-7 and TLR-8) are the major mechanistic targets for IMQ^7^, as its ligation activates the transcription mediator; NF-κβ, by the MyD88 pathway^8^. This activation of NF- κβ, in turn, escalates the genesis of several pro-inflammatory cytokines including tissue necrosis factor-α (TNF-α) and various interleukins such as IL-1β, IL-6, IL-17A, and the list goes on. Not limited to the innate immune system response of the body, IMQ, through MyD88-dependent IRF pathway, also orchestrate IFN-γ production and stimulate infiltration of cytotoxic CD8^+^ T-cells^3^. This amplification of the immune system enables IMQ to be helpful in cutaneous malignancies, viral infections, and as an adjuvant (exert response via TCI) for immunization. To sum up, the IMQ’s therapeutic activity was mainly mediated by optimal activation of MyD88 dependent NF-κβ activation, IFN-γ production and recruitment of Cytotoxic CD8^+^ T-cells and NK-cells^9,10^.

**Figure 1:**
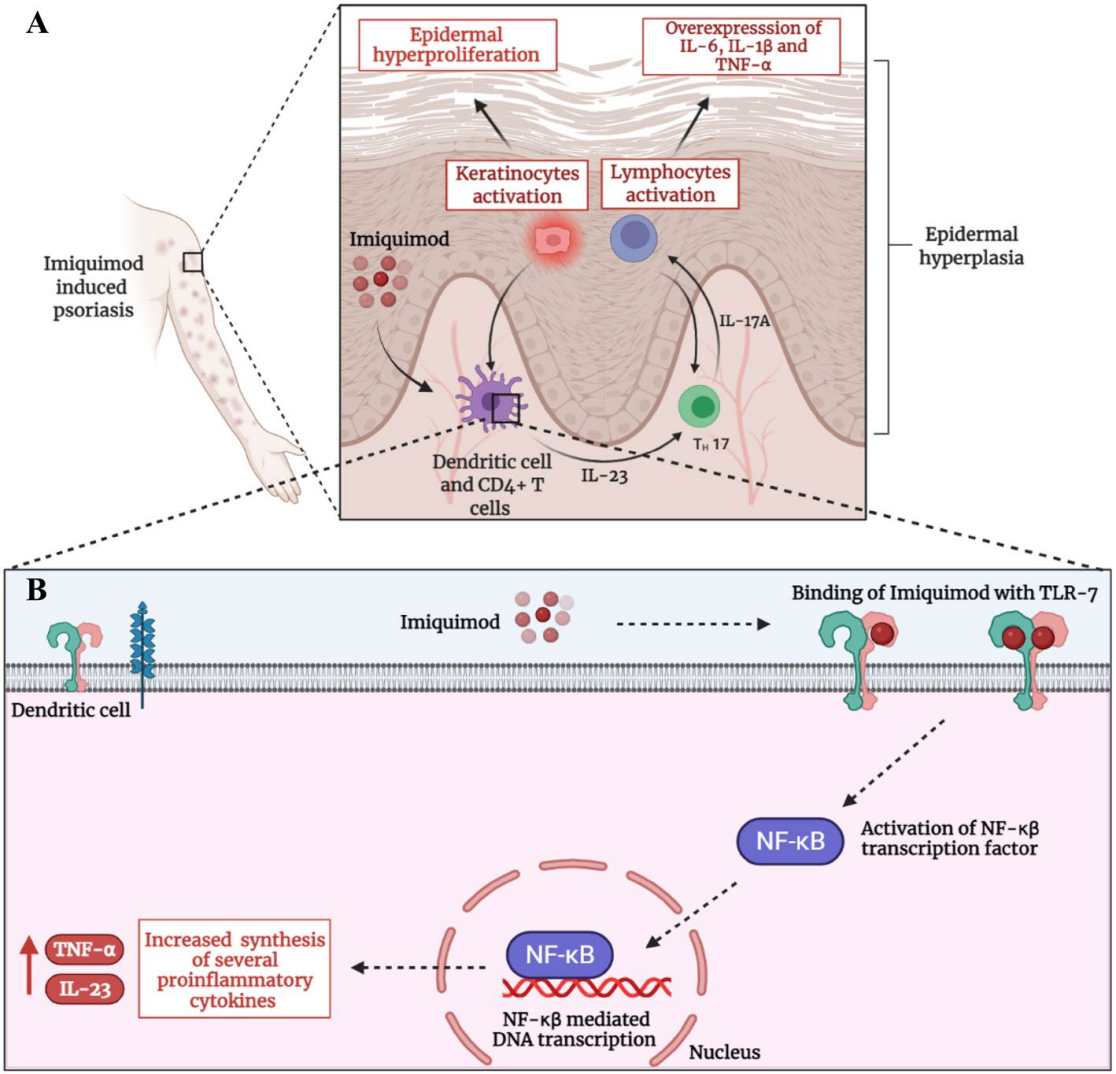
(**A**) Mechanistic illustration of IMQ induced psoriasis. (**B**) IMQ induced hyper-stimulation of NF-κβ and hyper-production of pro-inflammatory cytokines in dendritic cells. Created with BioRender.

Unfortunately, the overexpression of the TLR-7 dependent and independent pathways by ALDARA contributes to the emergence and manifestation of plaque-like psoriatic lesions^11^. Myriad published case reports have proven the fact that ALDARA, utilized for management of AK and BCC, can cause psoriasiform epidermal hyperplasia along with epidermal and dermal infiltration of inflammatory cells. Furthermore, complete resolution of lesions, following the treatment with anti-psoriatic drugs (Tacrolimus and Clobetasol), has also confirmed the possible toxicity of IMQ^12,13^. Nowadays, IMQ in the form of ALDARA is being utilized as a chemical inducer for the development of the psoriatic animal model to explore, investigate and assess the anti-psoriatic potential of newer agents^14–16^. Mechanistically, the IMQ induced psoriasiform lesions manifest by the overactivation of TLR-7 receptor expressed by the cutaneous dendritic cells^17^. In addition to the earlier stated immune response, the activation of these receptors also causes overproduction of IL-23 and TNF-α (via NF-κβ). The overexpressed IL-23 stimulates the activation of TH-17 lymphocytes which, in turn, promote the biosynthesis of IL-17A. TNF-α, together with IL-17A, effectuate keratinocytes hyperproliferation and release of other pro-inflammatory mediators- IL-1β, IL-6, IL-10, IL-17 and TNF-α. These absurd levels of many cytokines trigger neutrophils and helper CD4^+^ T-cells recruitment in the epidermis. All these events, ultimately precipitate the major pathological defects of psoriasis i.e. epidermal hyperplasia (hyperkeratosis) and acanthosis^18,19^. This mechanism is illustrated graphically in Fig. 1A,B. Furthermore, IMQ marketed formulation ALDARA, also exhibits cutaneous inflammation by TLR-7 independent pathway by activation of inflammasomes in the epidermis which stimulates the keratinocytes death as well as fibroblasts and IL-17A mediated hyperproliferation of keratinocytes^20^. This will also augment the TLR-7 activity on helper CD4^+^ T-cells and worsen the NF-κβ mediated immune activity as a feedback response via IL-1β pro-inflammatory cytokine^21^.

The physicochemical properties of IMQ are also considered to be a major reason behind its use-limiting toxic effects. Having a LogP-value of 2.6, the lipophilic IMQ is initially retained in the stratum corneum (SC) layer of the skin. This causes the desquamation of the SC. Following the eruption of the upper skin layer, IMQ undergoes significant permeation into the epidermis as well as in the dermis. However, due to minimal aqueous solubility, IMQ gets accumulated in cutaneous layers and is unable to permeate in the systemic circulation, significantly^22^. This excessive accumulation of IMQ in the skin ultimately triggers an abnormal immune response leading to psoriasis, by the pathway stated earlier. Employment of nanotechnology-based techniques; designing of drug-laden nanoparticles (nano-formulations), is considered to be an effective approach to prune away the drug-associated toxicity^23^. In the last decade, several scientific explorations have been conducted to minimize drug toxicity by fabricating their nano-formulations- liposomes, polymeric nanoparticles, Solid Lipid Nanoparticles (SLNs), and Nanostructured Lipid Carriers (NLCs), etc.^23–25^. Despite the broad anti-fungal spectrum of Amphotericin-B, its nephrotoxicity and infusion-related side effects halted its clinical use. Finally, scientists have designed liposomal amphotericin-B with a highly improved safety profile^23,26,27^. Similarly, the nano-formulations of myriad drugs including Miltefosine^28^, sirolimus^29^, doxorubicin^30^, and other anti-neoplastic agents have been developed in order to gain superior safety to the counterpart drugs. IMQ nano-formulation would possibly impart a hydrophilic character to it, which will equilibrate its cutaneous accumulation and increase its systemic flux^31^.

Therefore, in this study, we aimed to fabricate IMQ-nanotransethosomes (IMQ-nTES), incorporate them into the chitosan gel (IMQ-nTES gel), and characterize them on the basis of particle characterization, IMQ entrapment, in vitro drug release and ex vivo sin permeation and retention assay. Furthermore, IMQ-nTES gel was compared with ALDARA with respect to cutaneous toxicity in BALB/c mice. For extensive comparison of safety, several studies including psoriatic area severity index (PASI) scoring, Masson’s trichome, and H&E stained histopathology analysis, and quantification of the epidermal thickness of skin and ear as well as ear pinnae thickness, have been carried out. Furthermore, the immune response of both groups (ALDARA and IMQ-nTES gel treated group), in addition to the control group, was conducted by quantification of IL-6, IL-1β, TNF-α, IFN-γ and IL-17A. We have also assessed the immune system overexpression using immunohistochemistry (IHC) to assess NF-κβ expression and cutaneous CD4^+^ as well as CD8^+^ T-cells quantification using flowcytometry.

## Materials and methods

### Materials

Imiquimod with 99% purity, ethanol, and TWEEN-80, were acquired from Sigma Aldrich (Germany). Phospholipon 90G (PL90G) was gifted by Lipoid GmbH (Ludwigshafen, Germany). Imiquimod 5% marketed cream, ALDARA (MEDA pharmaceuticals, New Jersey, USA), was purchased from the local market.

### Animals

Six weeks old, male Sprague Dawley rats, weighing 180 ± 20 g, were utilized to perform ex vivo skin permeation and deposition study as well as in vivo skin irritation study. Furthermore, for in vivo cutaneous toxicity assessment, BALB/c mice were purchased from the National Institute of Health, Pakistan. All the mice, 6–8 weeks old, were weighed about 25 ± 5 g. Animals were kept and acclimatized under optimal temperature and humidity conditions in the animal house of Quaid-i-Azam University, Islamabad Pakistan. Moreover, all the animal experimentations were conducted according to the approved ethical protocols (BEC-FBS-QAU2021-316) adopted from the NIH guidelines. Beside this, all methods are reported in accordance with ARRIVE guidelines. Moreover, the animals were stipulated with a standard diet, clean drinking water, and a 12/12 h light/dark cycle.

### Preparation of IMQ-nTES

Fabrication of IMQ-nTES was conducted by cold method already reported by Abdulbaqi et al.^32^, with some modifications. Succinctly, the organic phase comprising of ethanol was prepared by dissolving IMQ and PL90G in it. This was maintained at 30 °C with continuous stirring at 700–1200 rpm. The water, containing Edge Activator (EA) (TWEEN-80), was instilled dropwise, at the rate of 10 drops/min, in an already prepared organic phase. The clear organic phase was eventually turned hazy indicating the development of TES. The vial containing IMQ-nTES were remained at continuous stirring for additional 30 min (at the same temperature) followed by extrusion with 0.22 μm nylon syringe filter (Gilson, USA), to attain desired vesicle size and poly dispersibility index (PDI) of IMQ-nTES^32^.

### Optimization of IMQ-nTES

Through preliminary experiments, different potentially influencing parameters of IMQ-nTES were ascertained. TWEEN -80, as an (EA), has been selected to design IMQ-nTES. Furthermore, 2^3^ full factorial design was employed for statistical optimization (using DESIGN EXPERT software ver. 12) of IMQ-nTES, in order to evaluate the effect of independent factors including PL90G and TWEEN -80 concentration (conc.), ethanol conc., and stirring rate on various response factors^32^. The major independent variables and response factors were studied for optimization and are stated in Table 1.

**Table 1:** Independent variables and response factors opted for optimization of IMQ-nTES.

Independent variables	Levels analyzed	
	Low level (− 1)	High level (+ 1)
**X**_**1**_: PL90G:EA conc. (mg)	75:25	95:5
**X**_**2**_: ethanol conc. (% v/v)	20	40
**X**_**3**_: stirring rate (rpm)	700	1200

Response factors (dependent)	Desirable response	
Y_1_: vesicle size (nm)	Minimize	
Y_2_: poly-dispersibility	Minimize	
Y_3_: entrapment efficiency (%)	Maximize	

### Vesicle (V.) size and poly dispersibility analysis of IMQ-nTES

The mean vesicle size (V. size) and PDI of IMQ-nTES were determined at 25 ± 1 °C by using Dynamic light scattering (DLS) Zetasizer Nano S-equipment (MALVERN ZETASIZER, MALVERN, UK) and data was analyzed by the Malvern Software^33^. For analysis, sample preparation was done by diluting 20 μl of IMQ-nTES dispersion in distilled water^34^.

### TEM analysis for IMQ-nTES vesicular morphology

The external morphology of designed IMQ-nTES vesicles was assessed using Transmission electron microscopy (TEM) (Make: Hitachi Ltd). IMQ-nTES droplet was placed on a carbon glazed copper grid of TEM, operated at 100 kV voltage. Phosphotungstic solution (2%) was employed, prior to the analysis, for negative staining of the sample film^35,36^.

### IMQ entrapment (%EE) within IMQ-nTES

According to the previously reported “Indirect” method, the percent entrapment efficiency of IMQ in IMQ-nTES was determined^37,38^. Briefly, 1 ml of IMQ-nTES dispersion was centrifuged (Model: Z216 MK, Make: HERMLE GmbH, Germany) at 13,500 rpm for 90 min at room temperature. Then, the clear supernatant was removed from the centrifuged dispersion and analyzed with UV-spectrophotometer (Model: HALO DB 20, Make: Dynamica, UK) at a wavelength of 245 nm^39^. Furthermore, the above mentioned mathematical formula was employed for the determination of entrapment efficiency,

$$\%EE\,\, of\,\, IMQ= \frac{Amount\,\, of \,\,entrapped\,\, IMQ}{Total\,\, amount\,\, of \,\,IMQ\,\, incorporated\,\, inside\,\, IMQ-nTES}\times 100$$

### Drug-excipients compatibility assessment using FTIR spectroscopy

For FTIR analysis, the characteristic peaks of plain IMQ powder, lipid (PL90G), their physical mixture, and the lyophilized powder of optimized IMQ-nTES were determined and compared using FTIR spectrophotometer (Model: FTIR-8300, Make: Shimadzu, Japan). The optimized IMQ-nTES formulation was centrifuged to collect its pallets, The pallets were, then, re-dispersed in a cryoprotectant (Mannitol 5% w/v). Finally, the mixture was subjected to primary and secondary drying with a lyophilizer (Model: Alpha 1-2 LD plus, Make: CHRIST lab, Germany)^40,41^.

### Perpetration of IMQ-nTES gel

Low molecular weight chitosan, a linear polysaccharide, was used as a gelling agent to formulate the IMQ-nTES loaded hydrogel. Briefly, 2–3% chitosan was incorporated into 1% acetic acid solution to prepare hydrogel. Along the way, the designed nanotransethosomes (20% w/v) were mixed with erstwhile designed blank hydrogel, under continuous stirring^42^.

### Physicochemical and rheological assessment of IMQ-nTES gel

Formulated IMQ-nTES gel was subjected to visual assessment for determination of physical texture. Moreover, the pH of IMQ-nTES gel was assessed using a pH meter (Model: INOLAB pH7110, Make: Xylem analytics, Germany). Viscosity, another important parameter of gel systems, was also assessed at various shear rates using a Brookfield viscometer (Model: DV3T, Make: Brookfield engineering, USA) at room temperature (25 ± 0.5 °C)^34,42,43^.

### In vitro IMQ release profile under skin simulated conditions

In vitro drug release from simple drug solutions, drug-loaded TES, and drug-loaded TES gel were determined using a methodology devised by Dar et al.,^44^. Shortly, all the three experimental entities were separately placed inside a dialysis bag in a water bath shaker containing PBS as a release media (maintained at 37.5 ± 1.0 °C). The release studies were assessed at pH 5.5 and pH 7.4. At multiple time intervals, 1 ml of aliquots were taken and analyzed, followed by the replacement with an equal volume of fresh buffer. The drug release data was ultimately run through a DD solver to evaluate the drug release coinciding with different pharmacokinetic models and to determine the best fit model.

### Ex vivo assessment of IMQ cutaneous permeation and deposition

For IMQ to be safer and efficacious, it should have resided inside deeper layers of the epidermis. Incorporation of IMQ inside the TES system will facilitate the breaching of the SC barrier and permeation of IMQ inside the deeper epidermis and dermis. In support of the claim, ex vivo skin permeation was conducted using a Franz diffusion cell having two compartments, maintained at 37.5 ± 1.0 °C. The excised skin sections (circular) were mounted between the donor and acceptor compartment containing IMQ-nTES and the PBS (having pH 7.4), respectively. The same setting was made for IMQ-nTES gel and plain IMQ-gel as well, in separate cells. Aliquots of 1 ml at certain periods (0, 1, 2, 4, 6, 12, and 24 h) were taken and conc. of IMQ was ascertained by determining absorbance using UV-spectrophotometer. Finally, the values were plotted against the time (at which the samples were taken) on GraphPad Prism^45^.

To determine the skin deposition of IMQ, a previously reported method was employed with slight modifications^46,47^. Shortly, the skin was detached from the cells and washed with PBS to remove the adhered formulations. By cellophane adhesive tape stripping method the SC was removed. The cellophane tapes strippings (approx. 18) were soaked in 70:30 methanol: ammonia acetate buffer (pH 4.5) for 8 h. Similarly, the stripped skin tissue (residual epidermis and dermis) was chopped and instilled in the erstwhile mentioned extraction media. Next, the probe sonication was conducted and the extract was subjected to extrusion by a 0.45 μm nylon filter. Finally, the IMQ was quantified in both; the removed SC tissue as well as residual epidermis and dermis using UV-spectrophotometry; by determining absorbance at 245 nm^47,48^.

### In vivo skin irritation study on Sprague Dawley rats and histopathology assessment post-application of IMQ-nTES gel

A skin irritation experiment was carried out to determine the non-irritant nature of IMQ-nTES gel. After randomized distribution of Sprague Dawley rats, three groups were made (n = 3). One group was administered with IMQ-nTES gel, and the rest of the two groups were positive control and negative control. Herein, the positive control group was treated with 1% Formalin solution. Primary dermal irritation index (PDII) was employed to determine the skin irritation potential in terms of cutaneous edema and erythema. Moreover, the H&E staining was also conducted to determine the histopathological variation^42,49^.

### In vivo cutaneous toxicity assessment

**Figure 2:**
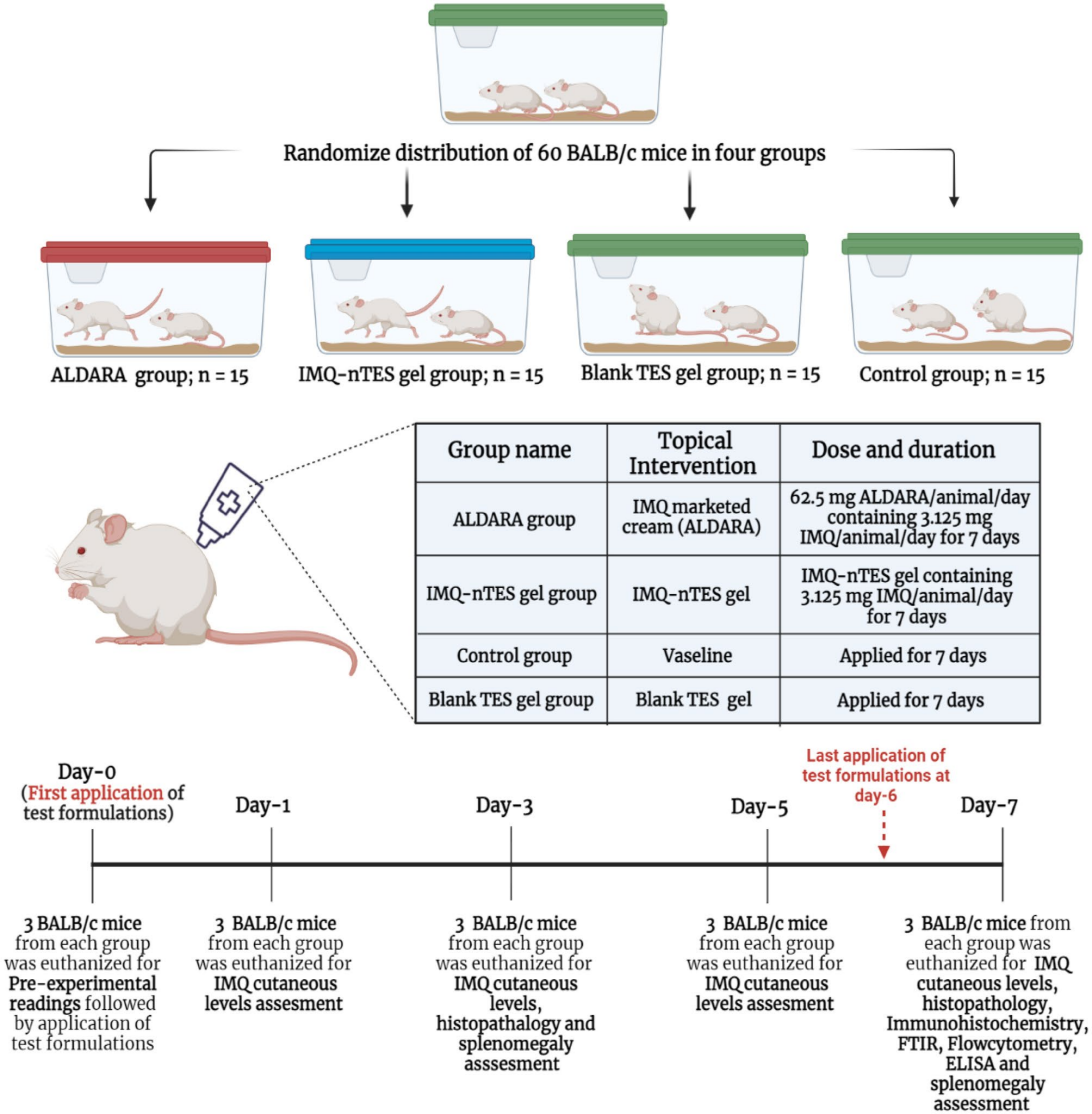
Experimental design for in vivo cutaneous toxicity assessment. Created with BioRender.

After acclimatization, sixty BALB/c mice were divided into four groups, each group was contained with 15 mice. Each animal of Group-1, (positive control group which was named as ALDARA group) was administered topically with 62.5 mg of ALDARA per animal per day (3.125 mg of IMQ/animal/day) on 2 cm × 3 cm shaved dorsal skin area for the uninterrupted duration of 7 days. Similarly, group-2 was designated as an experimental group (named as IMQ-nTES gel group) and applied topically with IMQ-nTES gel, delivering 3.125 mg of IMQ/animal/day, for consecutive 7 days. The group-3 was the negative control group (named as Control group) and was provided with the topical application of petroleum jelly (VASELINE). However, the blank TES gel was applied topically to the animals of group-4 (for a similar duration). Three mice, from each group, were sacrificed (before the application of experimental agents) for pre-experimental readings at day-0. In a similar fashion, three animals from each group were euthanized on day-1, 3, 5, and 7; for several aforementioned assays (also stated in Fig. 2);

- Day-1: IMQ cutaneous levels assessment,
- Day-3: IMQ cutaneous levels, histopathology and splenomegaly assessment,
- Day-5: IMQ cutaneous levels assessment,
- Day-7: IMQ cutaneous levels assessment, histopathology, FTIR for cutaneous structural alteration, Flow cytometry for CD4^+^ T-cells and CD8 + T-cells quantification, Immunohistochemistry for cutaneous expression of NF-κβ, ELISA for quantification of cutaneous IFN-γ and other cutaneous as well as systemic pro-inflammatory cytokines (IL-6, IL-1β, IL-17A, and TNF-α), splenomegaly, and C-reactive protein (CRP) assessment.

### Assessment of cutaneous imiquimod levels

Cutaneous IMQ conc. at day-1, day-3, day-5, and day-7 sacrificed mice of each of the four groups were assessed (n = 3, for each value). Initially, the removed skin was stripped using cellophane adhesive tape to remove the SC tissue. Following this, the stripped skin was chopped into small pieces and placed in a solvent system composed of methanol and ammonium acetate buffer (pH 4.5) in 7:3 v/v, for 24 h. Then, the samples were subjected to probe sonication thrice for 30 s, to achieve maximum extraction of IMQ located inside the skin layers. Finally, the extraction media^50^ was allowed to extrude through a 0.45 μm nylon filter and analyzed in UV-spectrophotometer for quantification^39,51^.

### PASI clinical scoring of BALB/c mice dorsal skin

IMQ induced cutaneous inflammatory response was assessed using a PASI clinical scoring. The shaved dorsal skin of all the mice belonging from the erstwhile mentioned four groups- ALDARA, IMQ-nTES gel, blank TES gel, and control group, were evaluated for three critical indicators and compared. These inflammatory indicators were erythema, scaling, and induration. Scoring was done consecutively for eight days and each parameter was assigned a specific score on the basis of visual examination (none = 0, mild = 1, mild = 2, moderately severe = 3 and highly severe = 4). Initially, n was equal to 15 and then, decreased subsequently to 3 (after dissection of 3 BALB/c mice from each group at day-0, day-1, day-3, and day-5)^52^.

### Effect on BALB/c mice ear thickness

To confirm that IMQ-nTES exhibit minimal cutaneous induration, the ear thickness of the IMQ-nTES gel treated group was compared with the ALDARA, blank TES gel, and control group using digital Vernier caliper (Make: Agar Scientific Ltd, UK). Formulations were applied topically on the left ear of each mouse of their respective groups, for 7 consecutive days, and results were compared. Initially, n was equal to 15 and then, reduced subsequently to 3 (after dissection of 3 BALB/c mice from each group at day-0, day-1, day-3, and day-5)^51^.

### Effect on cutaneous architecture and epidermal thickness

Dorsal skin tissues of day-0, day-3, and day-7 sacrificed BALB/c mice from IMQ-nTES gel, ALDARA, blank TES gel, and control group were fixed and stained with Masson’s trichome and H&E stain to evaluate in-depth cutaneous architectural variations (Yu et al., 2021). The stained slides were observed using an upright light microscope (Model: CX-41, Make: Olympus). Furthermore, the micrographs were evaluated in terms of epidermal thickness using ImageJ software (Version 1.52, Make: Java). The measured epidermal thickness from micrographs of all four groups was also compared statistically using the One-way ANOVA test^52,53^.

### ATR-FTIR analysis of skin to determine structure alteration

For cutaneous structural confirmation, in particular, for cutaneous lipid and proteinaceous contents, the excised skin of IMQ-nTES gel, ALDARA, blank TES gel, and control group were subjected to ATR-FTIR spectroscopic analysis. Briefly, the excised skin tissue was subjected to Diamond attenuated total reflectance-FTIR (Model: Cary 630 with diamond ATR module, Make: Agilent technologies) and measurements were taken from 4000–650 cm^−1^ wavenumber under the spectral resolution of 4 cm^−1^. The ATR-FTIR does not require any additional sample preparation techniques. The skin samples were mounted on the diamond ATR crystal with an application of slight pressure^42,54^.

### Flow cytometry analysis for quantification of cutaneous CD4^+^ and CD8^+^ T-cells

In several previously conducted studies, it was observed that the significant amplification of CD4^+^ T-cells has been observed in psoriatic skin^55,56^. However, the efficacy of IMQ in BCC and other medical indications is mainly governed by the cytotoxic CD8^+^ T-cells^9,10,57^. For quantification of CD4^+^ and CD8^+^ T-cells in skin samples of IMQ-nTES gel, ALDARA, blank TES gel, and control group, flowcytometric analysis was performed with protocols previously opted by Ali et al.,^58^. Initially, the mononuclear cells were incubated in a separate tube with an antibody named; anti-CD4-FTIC and anti-CD8-FTIC (Make: Exbio, Czech Republic), for 30 min at 25 °C, separately. The unattached antibodies were removed by washing the stained cell at 800×*g* for a duration of 10 min. Just prior to analysis, cell pallets were re-dispersed with phosphate buffer saline (PBS with pH = 7.4). Finally, the cells were analyzed on a BD FAC Scan Flow cytometer (Make: Becton Dickinson, United States). The FTIC-labelled antibody stained cells were attained at FL-1 channel in lower right quadrant.

### Immunohistochemistry based assessment of NF-κβ

The role of NF-κβ transcription factor in psoriasis has already been discussed in “Introduction”. Skin tissue samples from all four groups were subjected to immunohistochemistry analysis for NF-κβ detection. Skin samples were cut and placed over the positively charged slides which were then backed (at 65 °C) for the duration of 1 h. Then, the slides were de-paraffinized in xylene (twice for 10 min) and ethanol. Antigen retrieval was acquired by the utilization of the protein kinase and blocking with 5% normal goat serum (NGS). For NF- κβ detection, a primary polyclonal NF- κβ (anti-mouse) antibody was applied and incubated at 4 °C overnight. This was followed by the application of a secondary antibody, Avidin–Biotin complex, and finally stained with diaminobenzidine solution. The stained IHC slides were then examined under the Olympus CX-41 microscope^59^.

### ELISA for cutaneous cytokines quantification

Several inflammatory mediators including interleukin-6 (IL-6), IL-1β, IL-17A and TNF-α exhibit a decisive role in psoriatic pathogenesis^60^. The IFN-γ, however, stimulates the recruitment of cytotoxic CD8^+^ T-cells; essential for IMQ’s therapeutic response^9,10^. Excised skin from each of the four groups was homogenized and inserted into lysing buffer. The samples were then subjected to an ELISA kit (Make: Thermo Fisher scientific) for cytokine quantification to assess the inflammatory response comparatively^11,61^.

### Systemic inflammatory response assessment (Spleen size and mass, serum cytokines, and inflammatory markers)

The surge in various pro-inflammatory cytokines is usually observed in psoriasis, not only in the skin but also in the systemic circulation. Particularly, the levels of IL-6, IL-1β, and TNF-α significantly get elevated^62^. Moreover, a similar type of overloading of pro-inflammatory cytokines is generally observed with IMQ induced psoriatic lesions. Quantification of the above-mentioned cytokines was carried out using an ELISA kit (Make: Thermo Fisher scientific)^51^. Another systemic inflammatory indicator, the CRP, was also evaluated. Splenic mass and length of day-0, day-3, and day-7 sacrificed BALB/c mice, from all animal groups (IMQ-nTES gel, ALDARA, blank TES gel, and control) were determined and compared.

### Statistical analysis

All results in this experimental study are mentioned as Mean ± S.D. The IMQ-nTES formulation was optimized using the DESIGN EXPERT Ver. 12 (Stat-Ease, Minneapolis, US) and ANOVA was applied. All of the remaining data was analyzed using MS excel Ver. 365 (Microsoft, Washington, US) and GraphPad Prism Ver. 9 (GraphPad Software, San Diego, US). The major statistical test employed for analysis was Tukey’s multiple comparison test (one-way ANOVA) and Sidak’s multiple comparison test (Two-way ANOVA).

## Results

### Optimization of fabricated IMQ-nTES

**Figure 3:**
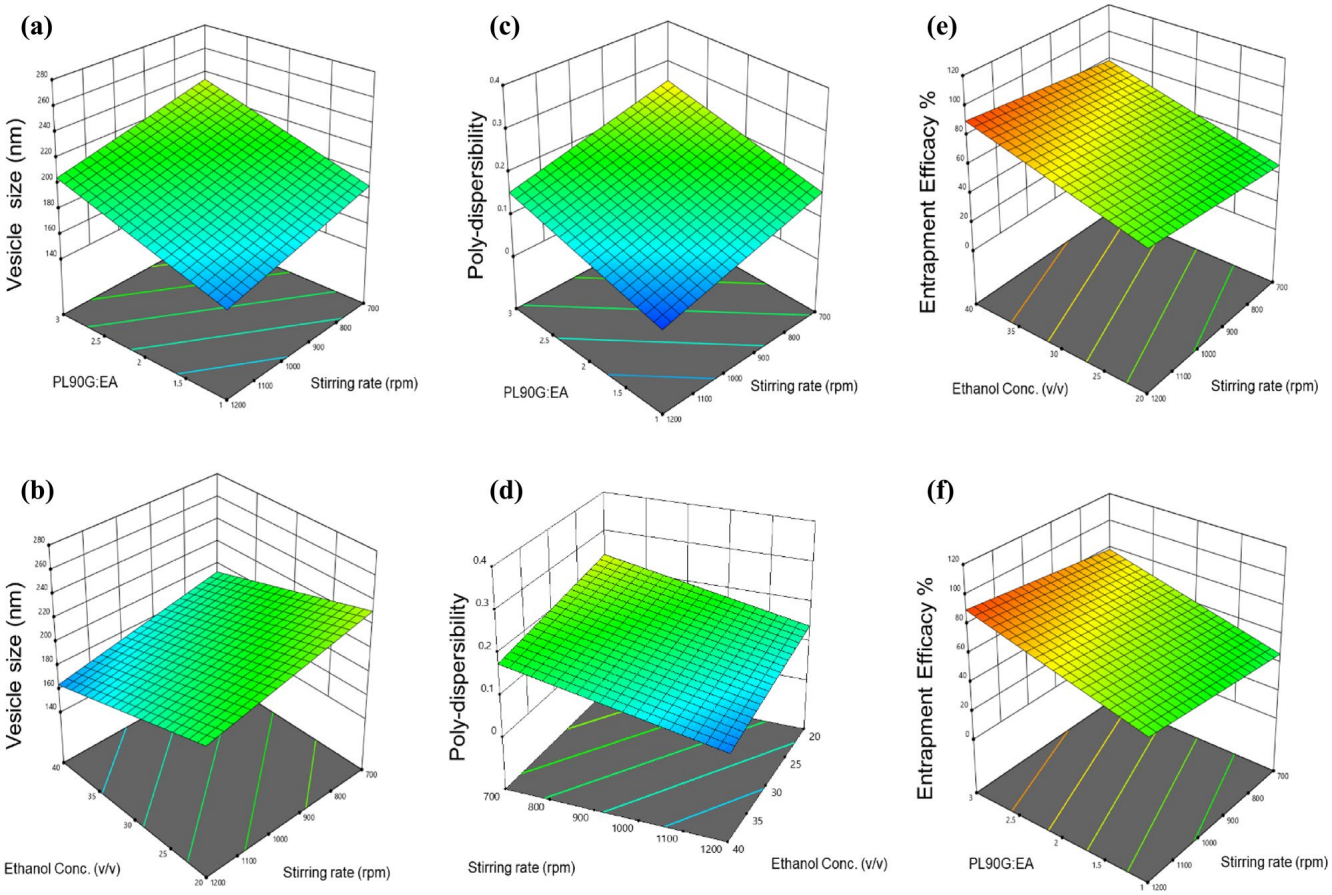
Surface (3-D) plots exhibiting the effects of independent variables upon the response factors; (**a**) effect of PL90G:EA ratio and stirring rate over the vesicle size; (**b**) effect of ethanol conc. and PL90G:EA on vesicle size; (**c**) effect of PL90G:EA ratio and stirring rate over the PDI; (**d**) effect of ethanol conc. and PL90G:EA on PDI. (**e**) Effect of ethanol conc. and stirring rate on the %EE; (**f**) effect of PL90G:EA and stirring rate on the %EE.

The cold method was employed to synthesize IMQ-nTES. The 2^3^-factorial design has generated 8 experimental runs and all the response factors were found to be in the desired range (stated in Table 2). Furthermore, the mean V. size, PDI, and %EE values lie between 146.03–272.2 nm, 0.049–0.205, and 10.50–95.4%, respectively. Model parameters, including R^2^, SD, and adequate precision are presented in Table 3. The effect of independent variables over the response factors has also been shown in form of three-dimensional plots (Fig. 3). The optimized response factors, using DESIGN EXPERT, were displayed as the V. size of 181.71 nm, PDI of 0.11, and 84.6%EE of IMQ. As a result, the formulation IMQ-nTES-3 was optimized and selected for further investigations.

**Table 2:** Composition, independent variables, and response factors of 8 IMQ-nTES formulations, generated by 2^3^ factorial design using DESIGN EXPERT.

Formulation	Independent variables			Response factors		
	X_1_	X_2_	X_3_	Y_1_	Y_2_	Y_3_
IMQ-nTES-1	95:5	20	1200	212.77 ± 2.73	0.205 ± 0.034	80.01 ± 2.11
IMQ-nTES-2	75:25	20	700	203.10 ± 1.23	0.191 ± 0.018	55.56 ± 2.04
IMQ-nTES-3	95:5	40	1200	192.4 ± 1.60	0.115 ± 0.008	91.05 ± 3.22
IMQ-nTES-4	95:5	20	700	272.20 ± 4.0	0.316 ± 0.017	89.60 ± 2.95
IMQ-nTES-5	75:25	40	700	175.46 ± 2.74	0.146 ± 0.005	68.15 ± 4.22
IMQ-nTES-6	75:25	20	1200	181.47 ± 1.8	0.049 ± 0.023	10.50 ± 0.83
IMQ-nTES-7	95:5	40	700	200.43 ± 0.66	0.181 ± 0.025	95.4 ± 2.77
IMQ-nTES-8	75:25	40	1200	146.03 ± 1.5	0.065 ± 0.021	53.21 ± 4.16

All values are mentioned as mean ± S.D (n = 3).

**Table 3:** Summary of applied 2^3^ factorial design, significant factors and optimized values of response factors.

Dependent Responses	R^2^	Predicted R^2^	Adjusted R^2^	Significant factors	Standard deviation	Adequate precision	Expected optimized parameters	Actual optimized parameters
Y_1_: particle size (nm)	0.9098	0.6392	0.8421	X_1_, X_2_, X_3_	14.45	10.8685	181.71	190.8
Y_2_: poly-dispersibility	0.8733	0.4930	0.7782	X_1_, X_3_	0.0403	8.9411	0.11	0.12
Y_3_: entrapment efficiency (%)	0.8760	0.5039	0.7830	X_1_, X_2_, X_3_	13.22	8.4634	84.6	91

#### Response of IMQ-nTES independent variables on V. size (Y_1_)

The V. size (Y_1_) of IMQ-nTES is a critical parameter towards the reduction of IMQ cutaneous toxicity. It was hypothesized that the nano vesicle would have better penetration potential as compared to the conventional dosage forms. The results generated by the 2^3^ factorial design has clearly demonstrated that all three independent variables exhibit significant effect over the size (Y_1_) of IMQ-nTES. It has been seen from the 3-D surface plot (Fig. 3a,b), with a reduction in lipid (PL90G) conc. and increase in EA conc., the V. size was significantly reduced and vice versa. Furthermore, the stirring rate was also directly influencing the V. size (Y_1_) of the particles. At 1200 rpm, small-sized particles were produced. As the stirring rate was reduced, the size of the particles was increased. The effect of ethanol was also similar to that of EA. The particles in nano-size were designed at the ethanol conc. of 40%v/v. Thus, with 75 and 25 mg respective quantity of PL90G and EA, 40%v/v ethanol, and a stirring rate of 1200 rpm, the size of the particles (Y_1_) was found to be 146.03 ± 1.5 nm, as observed with IMQ-nTES-8.

#### Response of IMQ-nTES independent variables on PDI (Y_2_)

As mentioned in Table 2, PDI (Y_2_) of all the designed formulations were found to be in the range 0.316 ± 0.017 to 0.049 ± 0.023. In order to achieve the smaller PDI (Y_2_) of IMQ-nTES vesicles, a low level of lipid (75 mg), high level of EA (25 mg), and 1200 rpm stirring rate was required, clearly seen in the graph presented in Fig. 3c,d. Both factors (PL90G: EA ratio and stirring rate) were expressed with the p-values of 0.032 and 0.025, respectively. Ethanol conc. has also influenced the PDI (Y_2_) of the nanosystem. The lower the conc. of ethanol, the higher the PDI (Y_2_) of IMQ-nTES was observed and vice versa. Thus, to attain the lower PDI, the IMQ-nTES was formulated with 75 and 25 mg of PL90G and EA, respectively, along with 40% v/v ethanol and 1200 rpm stirring rate, resulting in a PDI (Y_2_) value of 0.049 ± 0.023, as can be seen in case of the IMQ-nTES-6. In contrast to the IMQ-nTES-4, the PDI (Y_2_) of the formulation was found to be 0.316 ± 0.017 with the utilization of independent factors as follows; PL90G:EA in 95:5 mg with a stirring rate of 700 rpm.

#### Response of IMQ-nTES independent variables on %EE (Y3)

**Figure 4:**
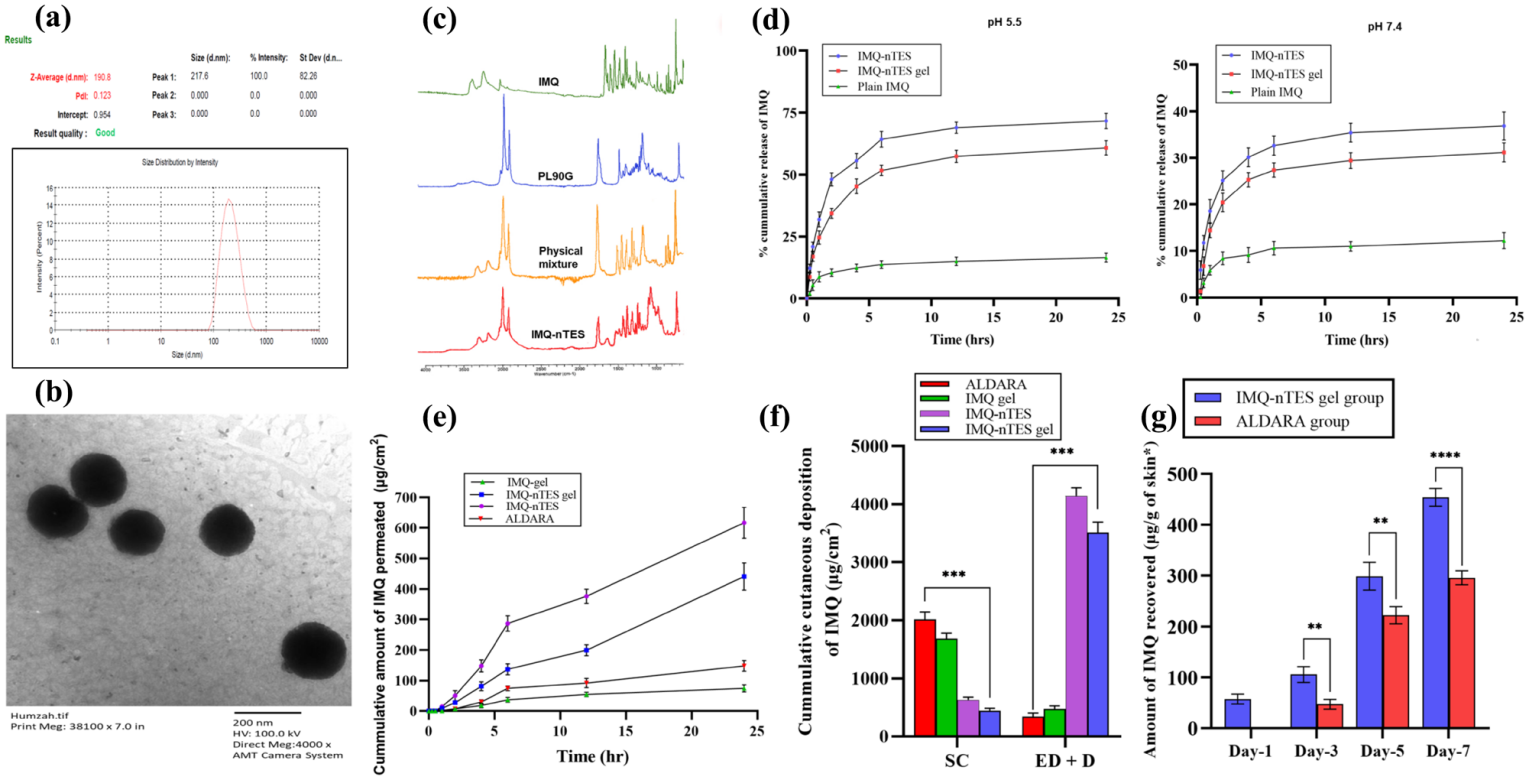
(**a**) Vesicle size and PDI of optimized IMQ-nTES. (**b**) TEM micrograph of optimized IMQ-nTES. (**c**) FTIR of IMQ-nTES and its component. (**d**) In vitro IMQ release pattern at pH 5.5 and pH 7.4. (**e**) Ex vivo skin permeation of IMQ. (**f**) Cumulative amount of IMQ deposition in rat skin. (**g**) IMQ amount recovered from stripped skin of disected BALB/c mice from IMQ-nTES gel and ALDARA group. Data is presented as mean ± S.D, n = 3; *p < 0.05, **p < 0.01, ***p < 0.001, ^ns^p > 0.05. *SC* stratum corneum, *ED* epidermis, *D* dermis.

Another important response factor, the %EE of IMQ (Y_3_), was mainly dependent upon two independent variables- the PL90G:EA ratio and the stirring rate. However, the effect of ethanol conc. exhibited a non-significant effect over %EE of IMQ (Y_3_), having a p-value of 0.123. The p-values for PL90G:EA and stirring rate were found to be 0.011 and 0.016, respectively. In other words, the %EE of IMQ (Y_3_) was increased with augmented PL90G conc., but the EA conc. displayed an opposite impact over %EE of IMQ (Y_3_). Higher IMQ content was entrapped at 90 and 5 mg of PL90G and EA, respectively (illustrated in Fig. 3e,f). In IMQ-nTES-7, the IMQ experience 95.4 ± 2.77 when the stirring rate was set at 700 rpm and the PL90G:EA conc. was 95:5 mg. The V. size and PDI results of optimized formulation (IMQ-nTES-3) are shown in Fig. 4a.

### TEM analysis of IMQ-nTES for surface morphology

The micrograph for TEM analysis illustrated in Fig. 4b, has shown the uniform size distribution as well as smooth and spherical surface morphology. Moreover, the results are also conforming the particle size analyzed by DLS.

### FTIR analysis for drug-excipient compatibility assessment

FTIR results have clearly demonstrated that there are chemical interaction exists among the IMQ, the PL90G, and the EA. All the characteristic functional group peaks of IMQ, PL90G, EA were found to be preserved in the prepared IMQ-nTES. The results are shown in Fig. 4c.

### Physicochemical characteristics of IMQ-nTES gel

Herein, low molecular weight (LMW) chitosan was selected as a gelling agent for the designing of IMQ-nTES gel owing to its intrinsic bio-adhesive nature and biological activities. Physically, the designed gel was milky whitish in color, clear in texture, and non-gritting (shown in Fig. S1). The pH of the designed IMQ-nTES gel was found to be (6.5 ± 0.3), indicative of its non-irritant nature because the pH is close to neutral. Furthermore, the rheology and flow properties of the IMQ-nTES gel have demonstrated the Non-Newtonian flow behavior- the viscosity of the gel decreased as the shear rate increased. This behavior eventually made the gel be applied with ease. Moreover, the percent spreadability was found to be 345.00 ± 5.00% indicating excellent spreadability. The viscosity graph of IMQ-nTES-gel is shown in the supplementary file (Fig. S1).

### In vitro release profile of IMQ

An in vitro release study of IMQ from IMQ-nTES and IMQ-nTES gel was conducted at both skin pH (5.5) and at blood pH (7.4) to elucidate its release pattern. At pH 5.5, 11.01 ± 1.54% and 14.31 ± 4.32% of the drug were released from IMQ suspension respectively at 4 h and 24 h. However, by incorporating IMQ inside nTES system, a significant surge in drug release was encountered. Briefly, the release of IMQ at 4 and 24 h was found to be 55.63 ± 3.01 and 74.31 ± 4.74%, respectively. Moreover, the release of IMQ from IMQ-nTES gel was slightly less owing to the presence of chitosan; a slight barrier to drug release, as depicted in Fig. 4d. The release from the IMQ-nTES gel at the same pH and increasing time duration was found to be 45.26 ± 3.04% and 60.80 ± 3.04%. The release pattern of IMQ from IMQ-nTES and IMQ-nTES gel was significantly increased and controlled when compared with the IMQ-suspension. Additionally, the drug release pattern from all the test formulations was also observed at blood pH (7.4). Notably, IMQ-nTES and IMQ-nTES gel have exhibited 31.17 ± 2.04% and 37.89 ± 4.58% IMQ release (at 24 h) in a controlled manner, contrary to the IMQ-suspension, which only release 12.20 ± 1.73% of IMQ during the 24 h release study. However, the less % cumulative release of IMQ from IMQ-nTES and IMQ-nTES gel was observed, in contrast to the % cumulative release encountered at 5.5 pH.

Upon the % cumulative release data of IMQ, the release kinetics models were applied and it was observed that both IMQ-nTES and IMQ-nTES gel mainly follow the Korsmeyer-Peppas model and exhibit drug release by a fickian diffusion (n values were less than 0.45 as shown in Table 4).

**Table 4:** Release kinetics modeling of IMQ-nTES and IMQ-nTES gel.

Formulation	pH	Zero order		First order		Korsmeyer–Peppas		Higuchi	
		R^2^	K_0_	R^2^	K_1_	R^2^	n	R^2^	K_H_
IMQ-nTES	5.5	− 0.8435	4.123	0.4644	0.1740	0.8946	0.284	0.5678	19.047
IMQ-nTES gel		− 0.6532	3.411	0.1747	0.089	0.8892	0.296	0.6231	15.685
IMQ-nTES	7.4	− 1.2181	2.189	− 0.7312	0.034	0.8748	0.261	0.4197	10.216
IMQ-nTES gel		− 0.4977	1.836	− 0.2237	0.026	0.8281	0.304	0.6097	8.465

### Ex vivo assessment of IMQ cutaneous permeation and deposition

The results of ex vivo IMQ cutaneous permeation and deposition assay are shown in Fig. 4e,f, and Table 5. As shown, after the application of ALDARA, SC has restricted the IMQ penetration in deeper epidermal layers, and displayed only 147.79 ± 17.63 μg/cm^2^ of IMQ cumulative transdermal flux. Contrary to that, IMQ-nTES and IMQ-nTES gel have displayed significantly improved results. Momentarily, results of skin deposition studies have shown that the cumulative amount deposited in SC tissue and deeper skin layer (inner epidermis and dermis) were 1684 ± 9.03 and 475.15 ± 52.86 μg/cm^2^, respectively after the application of IMQ-nTES and IMQ-nTES gel. These results respectively exhibited 8.3 folds and 5.5 folds of enhanced transdermal flux (IMQ) with IMQ-nTES and IMQ-nTES gel. Fortunately, the IMQ retention in the deeper epidermis and dermis was also improved markedly with the utilization of TES systems of IMQ. On the other hand, the IMQ was significantly retained in the SC tissue with ALDARA and IMQ-gel and only its small amount successfully reached deeper layers. In comparison to the ALDARA, IMQ-nTES and IMQ-nTES gel were presented with 11.92 and 10.15 times increased cumulative deposition of IMQ in deeper epidermis and dermis.

**Table 5:** Cumulative amount of IMQ deposited and permeated through the skin.

Formulation	Cumulative amount of IMQ permeated in 24 h (μg/cm^2^)	Cumulative amount of IMQ deposited in cutaneous layers after 24 h (μg/cm^2^)	
		Stratum corneum	Epidermis and dermis
IMQ-gel	74.56 ± 12.04	1684 ± 9.03	475.15 ± 52.86
ALDARA	147.79 ± 17.61	2015.71 ± 127.71	345.21 ± 59.04
IMQ-nTES	616.59 ± 51.12	633.99 ± 44.15	4145.56 ± 139.37
IMQ-nTES gel	410.36 ± 37.33	442.37 ± 44.88	3509.01 ± 182.64

All values are mentioned as a Mean ± S.D (n = 3).

### In vivo skin irritation study on Sprague Dawley rats and histopathology assessment post-application of IMQ-nTES gel

The IMQ-nTES gel was found to be non-irritant having a mean PDII of 0.9 which is significantly less (p-value < 0.05) than the mean PDII of standard irritant, 0.8% Formalin, having a value of 5.7. The histopathological micrographs have also shown that the tissue architecture of IMQ-nTES gel treated skin was comparable with that of normal skin. Furthermore, the leukocyte infiltration and destruction of epidermal tissues that were seen in the skin micrograph of 0.8% formalin were absent in the IMQ-nTES gel treated skin micrograph, as illustrated in Fig. S2.

### Cutaneous IMQ concentration assessment

Control and blank TES gel group was shown with no detection of IMQ throughout the 7-day study, however, it was quantified at day-1 in IMQ-nTES gel treated and at day-3 in ALDARA treated BALB/c mice. In the IMQ-nTES gel treated group, the IMQ exhibited 57.47 ± 9.57, 105.59 ± 15.48, 298.55 ± 15.49, and 453.82 ± 22.35, μg/g of striped mice skin at day-1, day-3, day-5, and day-7, respectively. In contrast, at day-1 IMQ was unable to be detected in ALDARA treated group. However, from day-3 to day-7, its level was elevated from 47.312 ± 9.70 to 295.37 ± 13.92 μg/g of striped mice skin. The results are presented graphically in Fig. 4g. At day-7. IMQ conc. was significantly (p < 0.0001) higher among IMQ-nTES treated mice, contrary to the rest of the three experimental groups.

### PASI clinical scoring of BALB/c mice dorsal skin for psoriatic severity assessment

**Figure 5:**
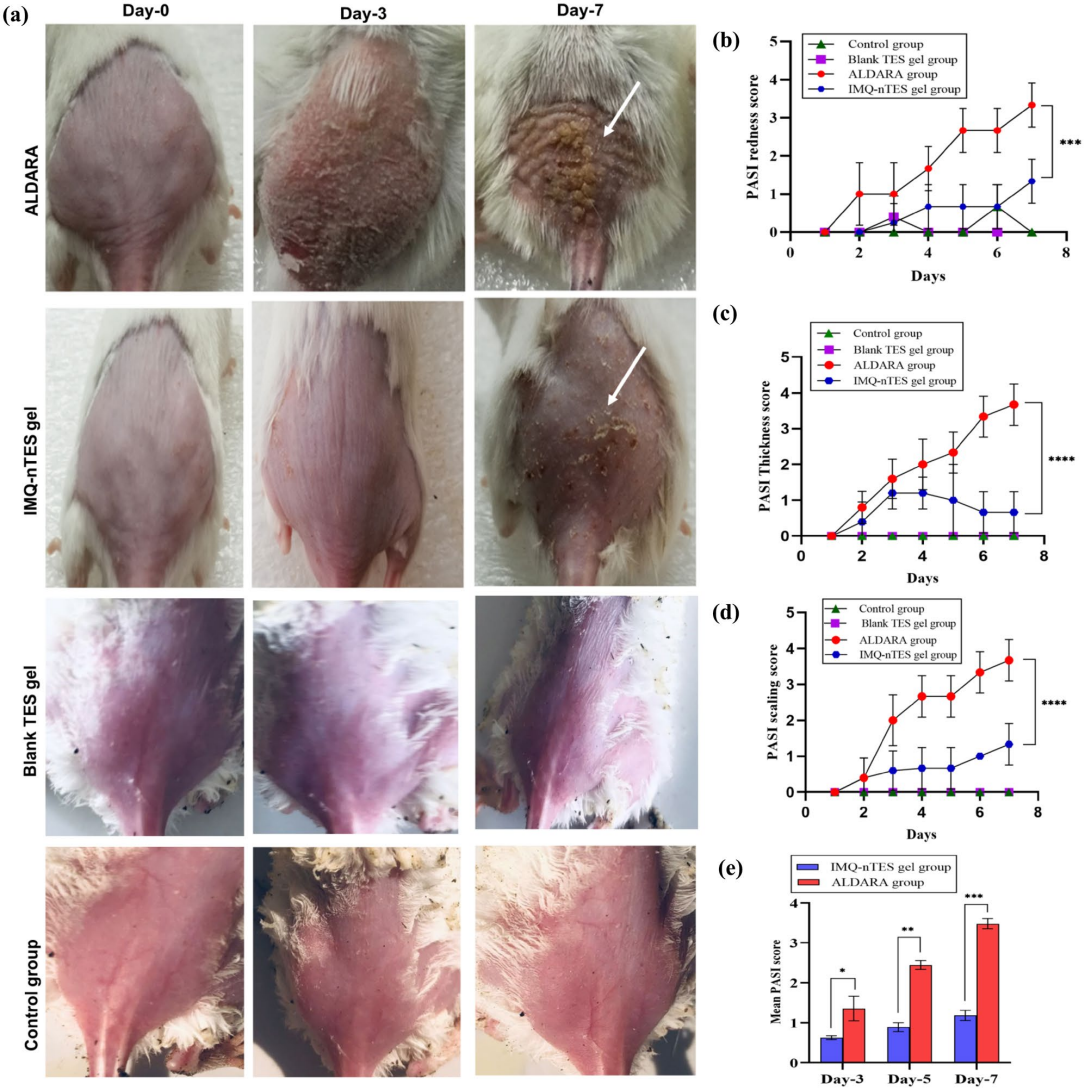
(**a**) IMQ induced cutaneous toxicity in ALDARA and IMQ-nTES gel group. (**b**) PASI redness score of all groups. (**c**) PASI skin thickness score of all four groups. (d) PASI scaling score of all four groups. (**e**) Mean PASI score. All the data is presented as mean ± S.D, n = 3; *p < 0.05, **p < 0.01, ***p < 0.001, ^ns^p > 0.05.

In Fig. 5a, animals, from all groups, are shown and only ALDARA group animals were manifested with severe psoriatic inflammatory signs. Individual PASI scores of redness, thickness, scaling and cumulative PASI score are presented graphically in Fig. 5b–e, respectively. Inflammatory signs, in both ALDARA and IMQ-nTES gel groups, were started manifesting from day-3. Furthermore, the cutaneous inflammation became severe and intense as the study progresses and on day-7 scores of all the three parameters were reached above score 3. However, in the IMQ-nTES gel group, the psoriatic signs were not intensified. Statistically, the disease severity is significantly (p < 0.0001) lower in the IMQ-nTES gel group as compared to the ALDARA group.

### Effect on BALB/c mice ear thickness- an indicator of epidermal hyperplasia

**Figure 6:**
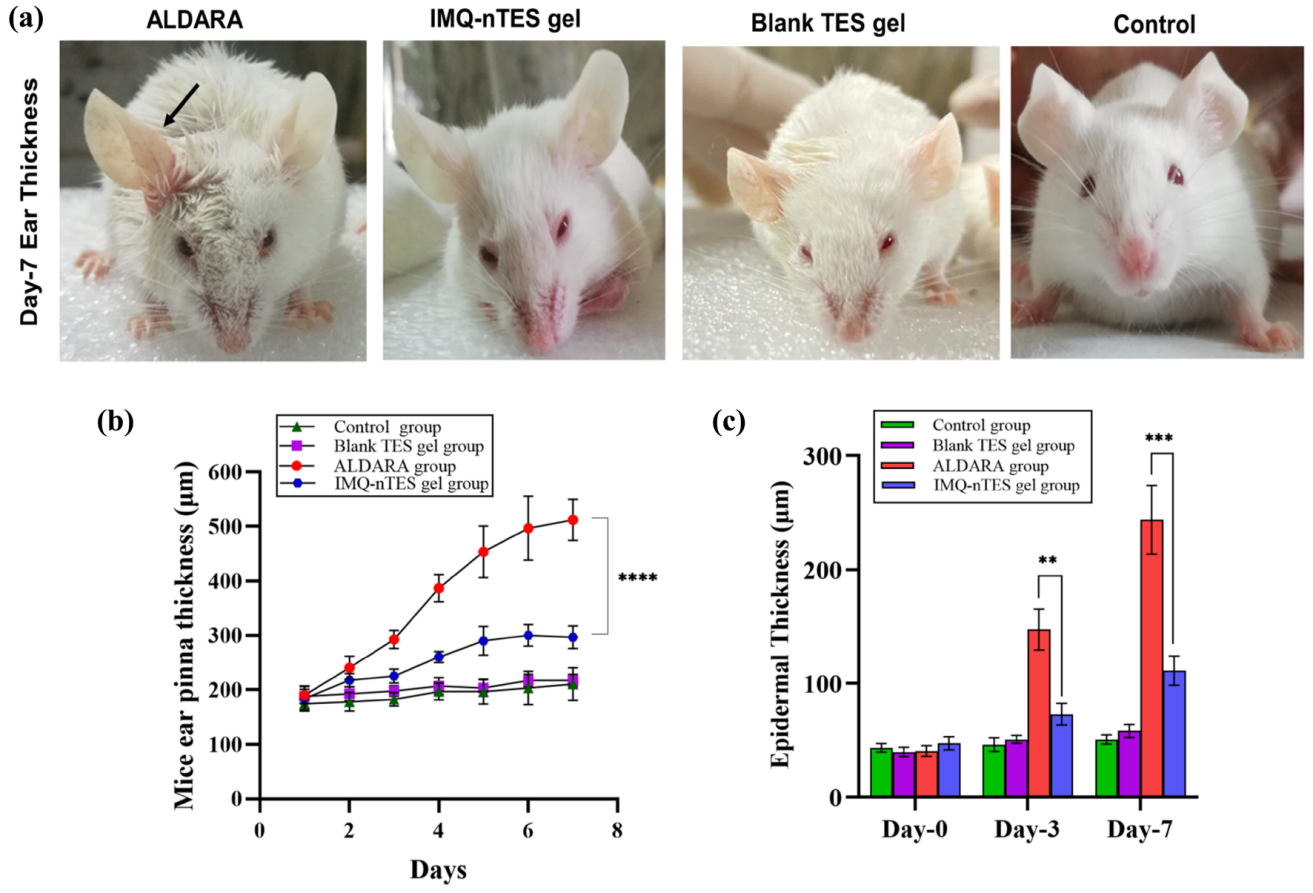
(**a**) Right ear pinnae enlargement (day-7) observed in all experimental groups (no enlargement was observed in Blank TES gel, IMQ-nTES gel and control group). (**b**) Mice right ear pinnae thickness observed in all groups. (**c**) Cutaneous epidermal thickness (in μm) of all experimental groups. All the data is presented as mean ± S.D, n = 3; *p < 0.05, **p < 0.01, ***p < 0.001, ^ns^p > 0.05.

Having a p-value less than 0.0001, the right ear pinnae thickness of BALB/c mice of the ALDARA group was higher (day-7 mean thickness = 511.7 ± 37.53 μm), with respect to the IMQ-nTES gel treated group having day-7 mean thickness of 296.7 ± 20.82. In contrast to the IMQ-nTES, our results clearly indicated the potential toxicity of ALDARA owing to its high hyperkeratosis potential (Fig. 6a,b).

### Influence over the cutaneous architecture and epidermal thickness

**Figure 7:**
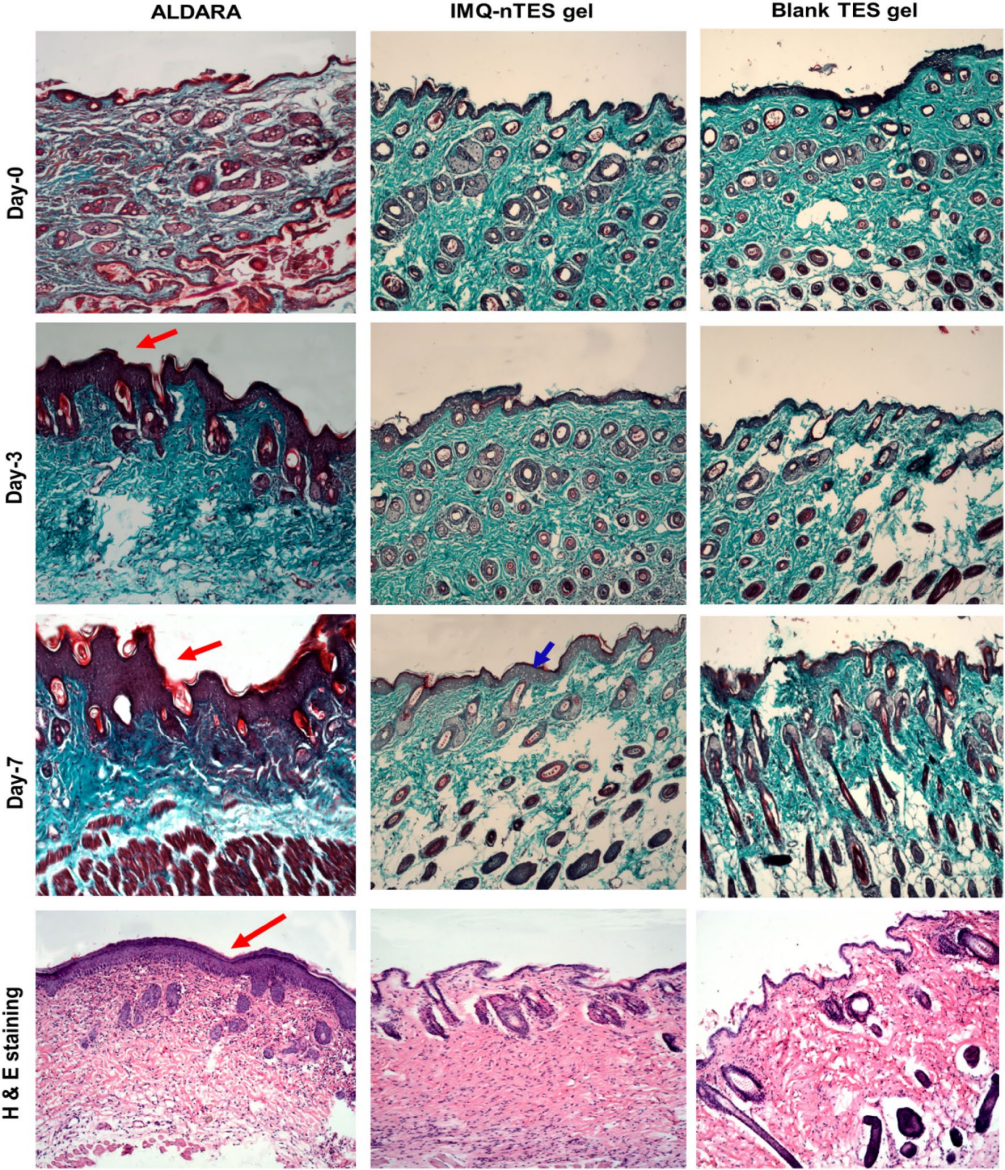
Trichome stained and H & E stained micrographs showing epidermal architecture of excised skin from dissected BALB/c mice from ALDARA, Blank TES gel and IMQ-nTES gel group.

Microscopic evaluation of trichome and H&E stained sections of BALB/c mice dorsal skin has shown a correlation with PASI disease severity scoring as well as ear epidermal hyperplasia (stated in “FTIR analysis for drug-excipient compatibility assessment” and “Physicochemical characteristics of IMQ-nTES gel”). The mean epidermal thickness of tissue micrographs from all animal groups is stated in Table 6 and graphically presented in Fig. 6c. Moreover, the micrographs of the trichome and H & E stained slides are presented in Fig. 7. Marked epidermal hyperproliferation was observed, (indicated with a red arrow in Fig. 7) on day-3 (147.32 ± 17.96 μm) which was further raised on day-7 (243.73 ± 30.25 μm), among the dissected animals of ALDARA group. These values were significantly lowered in the IMQ-nTES gel group dissected mice. At day-3, the epidermal thickness was observed as 72.89 ± 9.44 μm whereas at day-7 it was found to be 111.06 ± 12.97 μm. The control group, however, exhibits little or no change in the epidermal thickness.

**Table 6:** Epidermal thickness of ALDARA, IMQ-nTES gel, and control group dissected skin, evaluated using ImageJ software.

Group name	Epidermal thickness (μm)		
	Day-0	Day-3	Days-7
Control	43.22 ± 3.66	46.09 ± 6.10	50.66 ± 4.16
Blank-TES gel	39.49 ± 2.91	42.93 ± 4.42	42.38 ± 5.57
ALDARA	40.45 ± 4.64	147.32 ± 17.96	243.73 ± 30.25
IMQ-nTES gel	47.28 ± 5.84	72.89 ± 9.44	111.06 ± 12.97

All values are mentioned as a mean ± S.D (n = 3).

### ATR-FTIR analysis of skin to determine structure alteration

**Figure 8:**
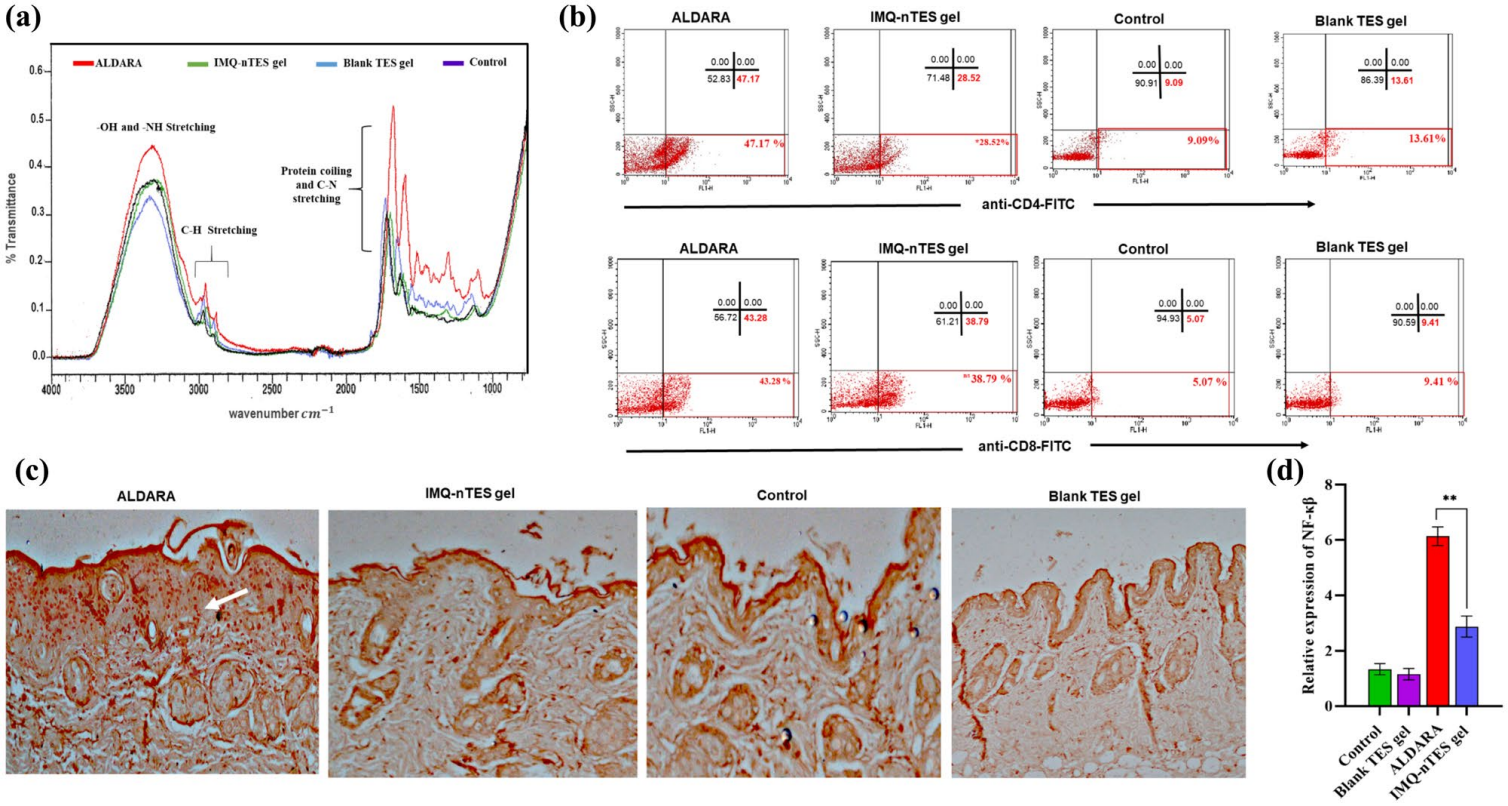
(**a**) FTIR curve of control group, Blank TES gel, IMQ-nTES gel and ALDARA treated skin (at day-7). (**b**) Flowcytometry for quantification of CD4^+^ T-cells and CD8^+^ T-cells population in skin of day-7 dissected mice of all groups. (**c**) IHC for NF-κβ expression in day-7 excised cutaneous tissue, with constitutive NF-κβ expression (round orange dots indicated by white arrow) in ALDARA treated skin. (**d**) Relative expression of NF-κβ in all experimental group. The data is presented as mean ± S.D. *p < 0.05, **p < 0.01, ***p < 0.001, ^ns^p > 0.05.

FTIR analysis of the excised skin was performed after ALDARA application for 7 days, which demonstrated the increased intensity of numerous peaks including peaks at 3310, 2970, 2870, 1650 and 1580 cm^−1^ (represented by the red plot in Fig. 8a), in comparison to the skin FTIR of the control group (normal skin) and blank TES gel group represented by purple and blue plot, respectively. These findings were, however, not much prominent with IMQ nTES gel treated skin. Skin FTIR results of all the four groups are represented in Fig. 8a and Table 7. These elevated peaks have shown higher lipid, cholesterol, and collagen (protein) content, as well as DNA glycosylation in the ALDARA, treated skin, a similar type of variation was observed in psoriatic skin, as indicated in the previously published report^63^. Furthermore, the secondary structure of cutaneous proteins was also modified as indicated by band position shift in ALDARA treated skin FTIR. With blank TES gel, no band shift or intensity increment was observed, indicating the absence of any structural modification in the skin. Similarly, the IMQ-nTES gel has not induced any major structural alteration in the skin as shown in the blue FTIR curve.

**Table 7:** ATR-FTIR skin analysis for determination of skin structure alteration.

Group	ATR-FTIR band position (cm^−1^)	ATR-FTIR Band Intensity	Bond stretching	Interpretation by ATR-FTIR band position and intensity
Control group	3290	0.36	–OH and –NH stretching	Normal protein and water content in skin
	2965	0.10	–CH (methyl and methylene) stretching of lipids and proteins	Normal protein and lipid content in skin
	2877	0.07		
	1690	0.30	C–N stretching, α-helix, and random coiling of proteins	The secondary structure of cutaneous proteins was preserved
	1610	0.18		
Blank TES gel	3290	0.36	–OH and –NH stretching	Normal protein and water content in skin
	2968	0.12	–CH (methyl and methylene) stretching of lipids and proteins	Normal protein and lipid content in skin
	2878	0.08		
	1695	0.29	C–N stretching, α-helix, and random coiling of proteins	The secondary structure of cutaneous proteins was preserved
	1620	0.17		
ALDARA	3310	0.45	–OH and –NH stretching	Higher band intensity was due to increment in water, protein, and glycosaminoglycans content in the skin
	2970	0.16	–CH (methyl and methylene) stretching of lipids and proteins	Increased intensity in –CH (methyl and methylene) stretching predicting the increased protein and lipid content in skin
	2870	0.10		
	1650	0.53	C–N stretching, α-helix, and random coiling of proteins	Alteration in the secondary structure of cutaneous proteins due to the band shifting with enhanced proteinaceous content
	1580	0.38		
IMQ-nTES gel	3306	0.33	–OH and –NH stretching	A slight elevation in cutaneous protein and water content
	2970	0.13	–CH (methyl and methylene) stretching of lipids and proteins	A slight increment in lipid content in skin
	2869	0.09		
	1699	0.33	C–N stretching, α-helix, and random coiling of proteins	The secondary structure of cutaneous proteins was not altered, however, a slight increment in protein content was observed
	1625	0.23		

### Flow cytometry analysis for quantification of cutaneous CD4^+^ and CD8^+^ T-cells

According to the illustrated results in Fig. 8b, with an application of ALDARA, there were a prominent surge in cutaneous CD4^+^ and CD8^+^ T cells, in comparison to the IMQ-nTES gel and control group. A Scatter plot of ALDARA treated skin has shown a significantly large amount of CD4^+^ and CD8^+^ expression in the lower right quadrant, as indicated by the red box. Fortunately, the CD4^+^ expression is lower (with p < 0.05) in the IMQ-nTES gel group. Upon quantification, it was found that CD4^+^ expression in ALDARA treated skin, IMQ-nTES gel treated skin and untreated skin (control group) was found to be 45.08 ± 2.09, 25.41 ± 3.11, and 10.31 ± 1.22, respectively (stated in Table 8). In addition to the IMQ-nTES gel group, the CD4^+^ expression with blank TES gel application was also comparable with the CD4^+^ expression of the control group. As far as CD8^+^ T-cells expression is concerned, the results of ALDARA treated skin and IMQ-nTES gel treated skin were found to be non-significant (p > 0.001) because ALDARA was presented with 43.52 ± 2.32% of CD8^+^ T-cells and IMQ-nTES gel has also stimulated the cellular immunity evident by 38.51 ± 2.90% of CD8^+^ T-cells. However, in the blank TES gel and control group, the percentage of CD8^+^ T-cells was significantly less, as seen in the scatter plot (Fig. 8b).

**Table 8:** Summary of cutaneous and systemic immune response in ALDARA, IMQ-nTES gel, and control group.

Group	Cutaneous inflammatory markers						Serum inflammatory markers			
	CD4^+^ cells (%)	CD8^+^ cells (%)	IL-6 (pg/ml)	IL-1β (pg/ml)	TNF-α (pg/ml)	IFN-γ (pg/ml)	CRP (mg/l)	IL-6 (pg/ml)	IL-1β (pg/ml)	TNF-α (pg/ml)
Control	10.31 ± 1.22	5.46 ± 1.36	27.08 ± 1.68	26.00 ± 2.65	36.48 ± 2.32	9.18 ± 1.40	6.09 ± 0.91	34.06 ± 1.02	41.03 ± 0.87	40.55 ± 1.37
Blank TES gel	12.14 ± 1.47	7.91 ± 1.56	27.75 ± 2.56	28.67 ± 2.52	35.26 ± 3.38	10.92 ± 1.377	5.71 ± 1.13	35.40 ± 2.98	40.37 ± 2.20	36.22 ± 3.28
ALDARA 5%	45.08 ± 2.09	43.52 ± 2.32	62.78 ± 3.13	90.37 ± 3.24	79.70 ± 2.52	45.21 ± 3.59	12.4 ± 1.03	74.4 ± 2.30	83.20 ± 2.13	81.77 ± 2.27
IMQ-nTES gel	25.41 ± 3.11	38.51 ± 2.90	31.77 ± 1.63	42.33 ± 3.51	47.98 ± 3.63	39.60 ± 4.45	7.03 ± 0.84	48.18 ± 1.61	57.46 ± 3.14	54.66 ± 3.14

All values are stated as mean ± S.D (n = 3).

### Immunohistochemistry based assessment of NF-κβ

The IHC micrographs, displaying the nuclear factor (NF-κβ) expression, are presented in Fig. 8c. Expression of nuclear factor (NF-κβ) was found to be elevated in skin samples applied with ALDARA cream, which is indicated by the white arrow in Fig. 8c. In contrast, IMQ-nTES gel treated group has shown a slight expression of transcription factor NF-κβ. In Fig. 8d, the relative expression of cutaneous NF-κβ is presented.

### ELISA for cutaneous cytokines quantification

**Figure 9:**
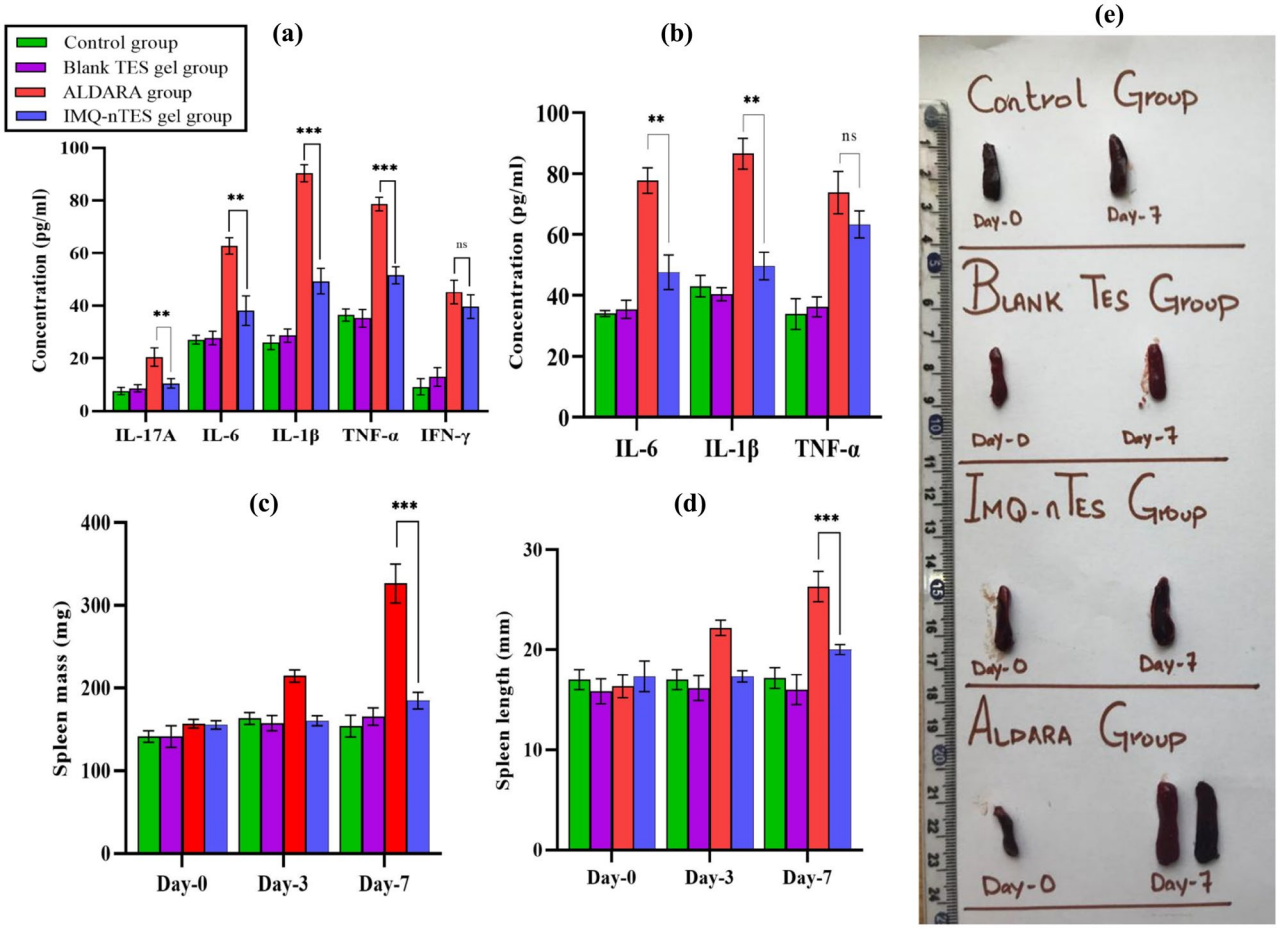
(**a**) Cutaneous cytokines level in ALDRA, IMQ-n TES gel, Blank TES gel and control group (at day-7). (**b**) Serum cytokines level in ALDARA, IMQ-n TES gel, blank TES gel and control group (at day-7). (**c**) Spleen mass in ALDARA, IMQ-n TES gel, blank TES gel and control group; (**d**) spleen length in ALDARA, IMQ-n TES gel, blank TES gel and control group; (**e**) pictorial representation of IMQ induced splenomegaly in in ALDARA and IMQ-n TES gel group. Data is presented as mean ± SD, for each value n = 3; *p < 0.05, **p < 0.01, ***p < 0.001, ^ns^p > 0.05.

Upon cutaneous application for 7 days, ALDARA treated skin displayed the highest conc. of IL-17A, IL-6, IL-1β, and TNF-α. IMQ-nTES gel application, however, presented significantly lower cytokines production, which was possibly due to reduced activation of inflammatory cells, as previously stated in “Influence over the cutaneous architecture and epidermal thickness”. In comparison to the control and blank TES gel, skin from the IMQ-nTES gel group revealed a higher conc. of IL-6, IL-1β, and TNF-α, in fact, a clear indicator of preserved IMQ immunomodulator activity. Surprisingly, IL-17A conc. is almost equal in both the IMQ-nTES gel group and the control group. IMQ-nTES gel treated skin also expressed the non-significantly variated conc. of INF-γ, in contrast to ALDARA treated skin. Cutaneous INF-γ expression in IMQ-nTES gel and ALDARA group was found to be 39.60 ± 4.45 and 45.21 ± 3.59 pg/ml, respectively. Cutaneous cytokine levels, in pg/ml, are illustrated graphically in Fig. 9a.

### Systemic inflammatory response assessment (Serum cytokines, spleen size, and mass and inflammatory markers)

With ALDARA, significantly elevated (p < 0.001) serum levels of IL-6 and IL-1β were observed with respect to IMQ-nTES gel, blank TES gel, and the control group. However, with IMQ-nTES gel non-significant (p > 0.05) elevation was observed, in contrast to blank TES gel and control group. In the case of serum TNF-α conc., there is a non-significant difference between ALDARA and IMQ-nTES gel groups. Elevation of TNF- α was observed in both groups. Results are presented graphically in Fig. 9b.

Another marker of systemic inflammation was assessed, the spleen enlargement in terms of size and length. IMQ-nTES gel was presented with a non-significant (p > 0.05) increment in splenic size and length, in comparison to the control group spleen. On day-7, with ALDARA, a significant elevation in spleen size and weight was observed- 26.33 ± 1.41 mm and 291.30 ± 28.34 mg, respectively. However, on day-7, the spleen of the IMQ-nTES gel group exhibited size and weight of 20.00 ± 0.5 mm and 178.33 ± 7.63 mg, respectively. All the results proclaiming enlargement in spleen size and mass (splenomegaly) are shown in Fig. 9c–e. As far as blood inflammatory markers (C-reactive protein and neutrophils) are concerned, C-reactive protein gave slight elevation in the ALDARA group, comparative to IMQ-nTES gel, blank TES gel, and control group. Systemic neutrophils were found to be similar. Results of serum cytokines level, cutaneous cytokines level, and systemic inflammatory markers are tabulated in Table 8.

## Discussion

The nanotechnology-based drug delivery approach is a worthwhile methodology to reduce drug-associated toxicities^64,65^. Previously, myriad nano-formulations have been designed including Amphotericin-B, Doxorubicin, Cilostazol, Daunorubicin, and Cytarabine to curtail the drug toxicity^23,66,67^. As mentioned in “Introduction”, actinic keratosis treatment with IMQ has emerged with the manifestation of de-novo psoriatic-like cutaneous lesions^13^. To lessen the psoriatic potential of IMQ, we have selected a nanotransethosomal system owing to its deformable nature and optimal cutaneous drug delivery^33,68^. In order to optimize IMQ-nTES and study the effect of independent variables (PL90G:EA conc., Ethanol conc. and stirring rate) over the response factors (V. size, PDI and %EE of IMQ), 2^3^-factorial design of DESIGN EXPERT software was employed^69^. The V. size of IMQ-nTES was found to be significantly affected by all the three independent variables, as stated in Table 3. With a decrement in PL90G conc. and increment in EA conc., the V. size and PDI of IMQ-nTES was reduced. Similarly, ethanol conc. was also associated with a reduction in V. size and PDI at higher ( +) levels. These findings are coherent with already published work by Moolakkadath et al.^70^. Furthermore, at a higher stirring rate lower V. size and PDI of IMQ-nTES were acquired, thus, it is also in accordance with the previously reported data^45^. Interestingly, for efficient cutaneous drug delivery, the V. size of the nanosystem should be in the range of 100–200 nm^45^. %EE of IMQ was another meaningful parameter that was believed to influence the selection of IMQ-nTES. It was observed that a higher IMQ amount was entrapped with lower EA and ethanol conc^33^. It could be because the higher EA and ethanol content generates pores in the lipid bilayer which could possibly cause the leakage of the entrapped drug^71^. However, the PL90G conc. has the opposite effect on the %EE of IMQ as higher lipid content holds more quantity of IMQ^70^. The optimized IMQ-nTES formulation showed excellent V. size, PDI, and %EE of IMQ.

Micrographs of TEM analysis of IMQ-nTES-3 were found to be in correlation with the V. size ascertained by DLS. Moreover, results have shown rounded shape, smooth and intact morphology of IMQ-nTES^65^. Drug-excipients incompatibility causes alteration in physicochemical properties as well as therapeutic nature of the formulation, therefore it is an important aspect to characterize and investigate^72^. FTIR spectroscopy was employed to assess this parameter and it was found that all the characteristics peak of IMQ and PL90G was preserved in both physical mixture as well as in IMQ-nTES. All the characteristics bonds were sustained and no peak shift was encountered. Hence, no chemical interaction was observed among the ingredients of IMQ-nTES^73^. Because of biocompatibility, and non-irritancy, and natural origin^74^, LMW chitosan was selected to develop IMQ-nTES gel and the whitish opaque IMQ-nTES gel has expressed a non-Newtonian flow indicating its easy application on the skin, with a percent spreadability of 345.00 ± 5.00%. The drug release pattern of IMQ-nTES and IMQ-nTES gel was assessed and compared with raw IMQ suspension. It was ascertained that the drug dissolution profile and release at pH 5.5 and 7.4 was significantly enhanced, in contrast to the IMQ-suspension. Owing to the weak basic nature of IMQ^75^ it was displayed with a better drug release profile at 5.5 pH, rather than at 7.4 pH^76^. Furthermore, the release profile has also predicted the sustained release pattern of IMQ from IMQ-nTES and IMQ-nTES gel, owing to the gel formation at the site of action^34,77^. Being lipophilic, IMQ is unable to secure significant accumulation in deep cutaneous tissues, in particular, the inner layers of the epidermis and the dermis. The distribution and retention of IMQ in the SC cells is the major limiting factor towards its minimal efficacy and toxicity^22,51,78^ In SC, the deposited amount of IMQ with plain IMQ gel and ALDARA were higher. The IMQ-nTES gel was found to be advantageous as it has provided significantly enhanced IMQ deposition in deeper epidermis and dermis i.e. 7.3 folds increased in comparison to the plain IMQ-gel and 10.15 folds increased in comparison to the ALDARA. Conclusively, the lipophilicity of IMQ is the major pretext for its reduced cutaneous penetration and higher conc. in SC. This issue was resolved by the employment of IMQ-nTES gel. Further, it exhibited that the designed IMQ-nTES gel can be used to compare for its cutaneous toxicity potential with ALDARA cream.

With an application of ALDARA, IMQ was not able to be quantified in stripped skin (at day-1) because IMQ permeation was halted by the SC barrier, as reported earlier^22^. The recovered amount was still lower on day-3. But on day-5 and day-7, the recovered levels of IMQ were increased by 221.99 ± 16.84 and 295.37 ± 13.92, correspondingly. Symptoms were also intensified from day-3 to day-7 (in the ALDARA group), mainly due to the dissemination of IMQ across the skin lipids and the PASI score of all three parameters surpassed the score 3. Interestingly, the recovered IMQ amount in the skin, followed by an application of IMQ-nTES gel, was significantly higher, in comparison to the erstwhile mentioned ALDARA group (shown graphically in Fig. 4g). Psoriatic lesions manifestation and PASI score were, however, significantly reduced as illustrated in Fig. 5a. Similarly, the right ear pinnae thickness was also comparable to the control (normal mice). From histopathology micrographs, indistinguishable findings were acquired. Marked inflamed hyperplastic epidermal layer can be seen in the ALDARA group on day-7 and it is significantly higher than IMQ-nTES gel treated/treated epidermis. The results of epidermal thickness in all three groups are mentioned erstwhile in “PASI clinical scoring of BALB/c mice dorsal skin for psoriatic severity assessment”. Despite the higher amounts of recovered IMQ from the striped cutaneous tissues of the IMQ-nTES gel group, the lower psoriatic potential was observed. The pretext of this finding is that the IMQ has entrapped inside the TES vesicles and released from the vesicle in a controlled manner. Thus, only the released drug was exposed to the cutaneous tissues which ultimately presented with significantly reduced cutaneous toxicity and PASI score^79^.

FTIR analysis curve of normal skin (control group) has displayed specific bands or peaks. Peaks at 1610 and 1690 cm^−1^ are the characteristic peaks for C-N stretching, α-helix, and random coiling of cutaneous proteins which depicts the secondary structures of proteins. However, peaks of 2877 and 3965 cm^−1^ are for lipid, cholesterol, and proteins methyl (C-H bond) stretching, while the 3290 cm^−1^ band is exhibiting -OH and -NH stretching of proteins, glycosaminoglycans, and water^54,80^. Previously, it was reported that in psoriatic skin several structural variations are usually encountered, in comparison to normal skin, including increment in skin lipid content, in particular the cholesterol. Furthermore, the structural proteins of skin; elastin and collagen, also surged in psoriasis, along with alteration in their secondary structure^63^. Results of FTIR analysis have clearly demonstrated all the erstwhile mentioned structural variations, evidenced by the increase in the intensity of peaks at 3310, 2970, 2873, 1650, and 1582 cm^−1^ in the red-colored plot (ALDARA) in Fig. 8a. Moreover, the peak shift (in ALDARA treated skin FTIR plot), from 1690 cm^−1^ to 1650 cm^−1^ and 1610 cm^−1^ to 1582 cm^−1^, demonstrated the alteration in coiling of cutaneous proteins. The control group plot (green color) is, in fact, a representative of normal skin FTIR where all such structural alterations are absent, having normal peak intensity. Similarly, the FTIR of blank TES gel group skin was also found to be coherent with skin FTIR of a control group, along with an absence of any structural alteration in the skin. However, in the IMQ-nTES gel treated skin FTIR plot, peaks at 3306, 1699, and 1625 cm^−1^ are very slightly affected in comparison to the ALDARA FTIR plot. Thus, FTIR results have demonstrated that the structural variation is significantly higher in the ALDARA applied to the skin, whereas, with IMQ-nTES gel treatment very little or no structural variations were encountered, in terms of increase in cholesterol, lipid, and proteinaceous content in the skin.

Interestingly, the pathogenesis of psoriasis lies upon dysregulation and overexpression of helper T-cells including CD3^+^ and CD4^+^ T-cells. In other words, amplification of helper CD4^+^ T-cells response was observed in psoriatic skin^55,56^. Thus, through flowcytometry, the cutaneous expression of CD4^+^ T-cells was quantified. Among all groups, ALDARA has shown maximum expression of CD4^+^ T cells, which is 45.08 ± 2.09%. Flowcytometry results; scatter plot in Fig. 8b, have shown high fluorescent intensity, FL1-H at the x-axis, in ALDARA treated skin. However, the incorporation of IMQ inside TES was found to minimize this unusual dysregulation of helper T-cells in the skin, as evidenced by only 25.41 ± 3.11% of the total cell content. Though, IMQ-nTES gel induces cutaneous helper T-cells activation but to a lesser extent than observed with ALDARA application. It has been reported that CD4^+^ T-cells are the continuous residents of normal mice skin as they are the main pillar of the cutaneous defense system. Moreover, they are in equilibrium with the circulating CD4^+^ T-cells. Roughly the quantity of normal skin resident CD4^+^ T-cells was five to six times less than that of infected or inflamed skin. In our study, the findings (percent CD4^+^ T-cells in normal skin/control) are in correlation with their stated results^81^. The therapeutics effect of IMQ against AK and cutaneous malignancies like BCC is chiefly governed by the cytotoxic CD8^+^ T-cells which were activated by optimal stimulation of TLR-7 mediated MyD88 dependent NF-κβ activation (non-constitutive NF-κβ production) and IFN-γ^9,10,57,82^. Our findings clearly show that the cutaneous recruitment of cytotoxic CD8^+^ T-cells is non-significantly less than ALDARA cream because, despite the reduced expression of NF-κβ, the activation and recruitment of cytotoxic CD8^+^ T-cells were agonized by IFN-γ. By this, we can claim that the efficacy of IMQ is preserved and unaffected by its incorporation inside the TES. With ALDARA, the activation of IMQ independent inflammation pathway (Inflammasomes) by one of its component isosteric acid, results in IL-17A and IL-1β production^20^. Moreover, IL-1β augments the TLR-7 activity on helper T-cells and worsens the NF-κβ mediated immune activity as a feedback response^21^. Furthermore, the constitutive production of NF-κβ is also the culprit for psoriatic pathogenesis as well as other anomalies^60,83^. This activation of the IMQ independent inflammation pathway was not observed with IMQ-nTES gel as evident by markedly reduced expression of constitutive NF-κβ, helper CD4^+^ T cells, IL-1β and IL-17A. Moreover, the overexpression of NF-κβ (constitutive NF-κβ) was also absent in IMQ-nTES treated group. Therefore, the cutaneous toxicity of IMQ-nTES is significantly less than that observed with ALDARA cream. Sustain release behavior and deeper cutaneous penetration of the IMQ-nTES gel system may also contribute to reduced cutaneous toxicity due to minimal exposure of IMQ to SC keratinocytes because IMQ also exerts direct damage to keratinocytes^84,85^.

According to the psoriatic inflammatory cascade, DCs and T-cells expression was increased which in turn has increased the synthesis of NF-κβ. As a result, NF-κβ hyperactive helper CD4^+^ T-cells caused overproduction of inflammatory cytokines which plays a pivotal role in psoriatic pathogenesis. Previously, it has been stated by various published reports that IMQ induced psoriatic skin is associated with marked elevation of interleukins (IL-6, IL-17A, and IL-1β) as well as TNF-α^11,19,86,87^. Maximum expression of NF-κβ was observed with ALDARA. IMQ-nTES gel was, however, found to be comparatively safer than conventional ALDARA cream, owing to optimal stimulation of cutaneous immune cells, non-constitutive NF-κβ, and cytokines synthesis. According to the study published by Li et al., the constitutive (overexpressed) NF-κβ has resulted in the pathogenesis of Hepatocellular carcinoma^83^. Basically, the NF-κβ can be beneficial as well as harmful and can be referred to as a double-edged sword. Its slight dysregulation or constative production can cause several pathologies due to the activation of myriad pro-inflammatory genes. Hence, for the therapeutic response, it should be expressed in a non-constitutive manner^88^.

IMQ-nTES gel skin, contrary to the control and blank TES gel group, manifest higher levels of IL-6, IL-1β, and TNF-α which is indicative of the fact that IMQ immunomodulator activity is completely preserved. IL-17A is a core cytokine in IMQ-induced psoriatic pathogenesis^11^. The IL-17A levels in IMQ-nTES gel, blank TES gel, and the control group was found to be low, as shown in Fig. 9a. This interesting finding can make IMQ-nTES gel a suitable alternate to ALDARA cream. MyD88-dependent IRF pathway stimulates the production of IFN-γ which possess a crucial role in the activation of cytotoxic T-cells and the therapeutics efficacy of IMQ. The results have shown a very minute reduction of cutaneous IFN-γ levels, in contrast to ALDARA cream. Another problem associated with conventional IMQ cream (ALDARA) is that it can also induce a systemic immune response, this finding was also specified in erstwhile published data^51^. Serum inflammatory cytokines (IL-6 and IL-1β) levels with IMQ-nTES gel application for 7 days were associated with minimal effect on the systemic immune response. However, serum TNF-α level was surprisingly elevated with IMQ-nTES gel. This was possibly due to IMQ intrinsic immune modulation ability, as skin and serum TNF-α levels were almost elevated to an equivalent extent. Spleen enlargement is an appropriate factor to gauge hyperactive systemic immune response as an enlarged spleen promotes the recruitment and amplification of several immune cells of the body^89^. IMQ-nTES gel has no significant influence over the spleen size and length and was almost similar to the control group, as illustrated in Fig. 9c–e. A surge in spleen length and weight with ALDARA however, showed that plain IMQ also triggers the systemic immune response which is undesirable. In a nutshell, the designed IMQ-nTES gel exhibited a better safety profile as it provides optimal activation of the cutaneous immune response, contrary to the ALDARA. Further investigations of IMQ-nTES gel on clinical grounds could make it a suitable alternate to the ALDARA.

## Conclusion

Imiquimod-loaded transethosomes (IMQ-nTES) were optimized using 2^3^ factorial design (DESIGN EXPERT) and the formulation IMQ-nTES-3 was selected because all the response factors were in the desired range. After characterization, an optimized formulation was incorporated in LMW chitosan gel to apply topically on the skin. The designed IMQ-nTES gel system was found to be superior to ALDARA in terms of reduced cutaneous toxicity potential. This assertion was supported by the 7-day in vivo comparative toxicity study on BALB/c mice which was further assessed on physical, histological, and molecular grounds. The absence of psoriatic lesions, lower PASI score, and non-significant increment in ear pinnae thickness were the suggestive facts towards improved safety profile of IMQ-nTES gel. Furthermore, non-significant epidermal hyperplasia was also observed with IMQ-nTES gel. Several indicators of cutaneous immune response (IL-6, IL-1β, IL-17A, TNF-α, IFN-γ and CD4^+^ as well as CD8^+^T-cells) and systemic immune response (Splenomegaly, serum cytokines, and CRP) were also determined, compared (with ALDARA) and manifested that IMQ-nTES gel could be of potential interest in terms of safety and efficacy, however, this candidate requires to be investigated for pre-clinical therapeutic efficacy evaluation.

## Supplementary Information

Supplementary Figures.

## Data Availability

All data generated or analysed during this study are included in this published article and its supplementary information files.
