# Supplementary material for: A cutback in Imiquimod cutaneous toxicity; comparative cutaneous toxicity analysis of Imiquimod nanotransethosomal gel with 5% marketed cream on the BALB/c mice
Source: Sci Rep. 2022 Aug 20;12:14244. doi: 10.1038/s41598-022-18671-1 (PMC9392762; doi:10.1038/s41598-022-18671-1)
Supplement: Supplementary file 1 — Supplementary Figures. [file 41598_2022_18671_MOESM1_ESM.docx]

**Supplementary file**

**A cutback in Imiquimod cutaneous toxicity; comparative cutaneous toxicity analysis of Imiquimod nanotransethosomal gel with 5% marketed cream on the BALB/c mice
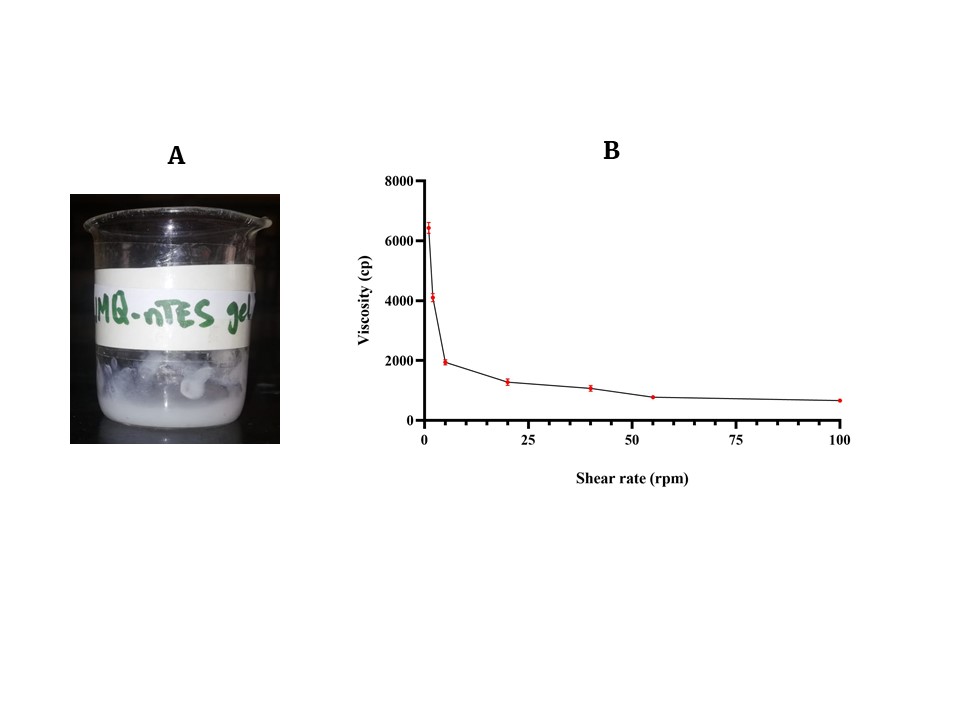
**

**Figure S1 [A] Physical appearance of IMQ-nTES gel. [B] Viscosity v/s shear rate curve of IMQ-nTES gel.**


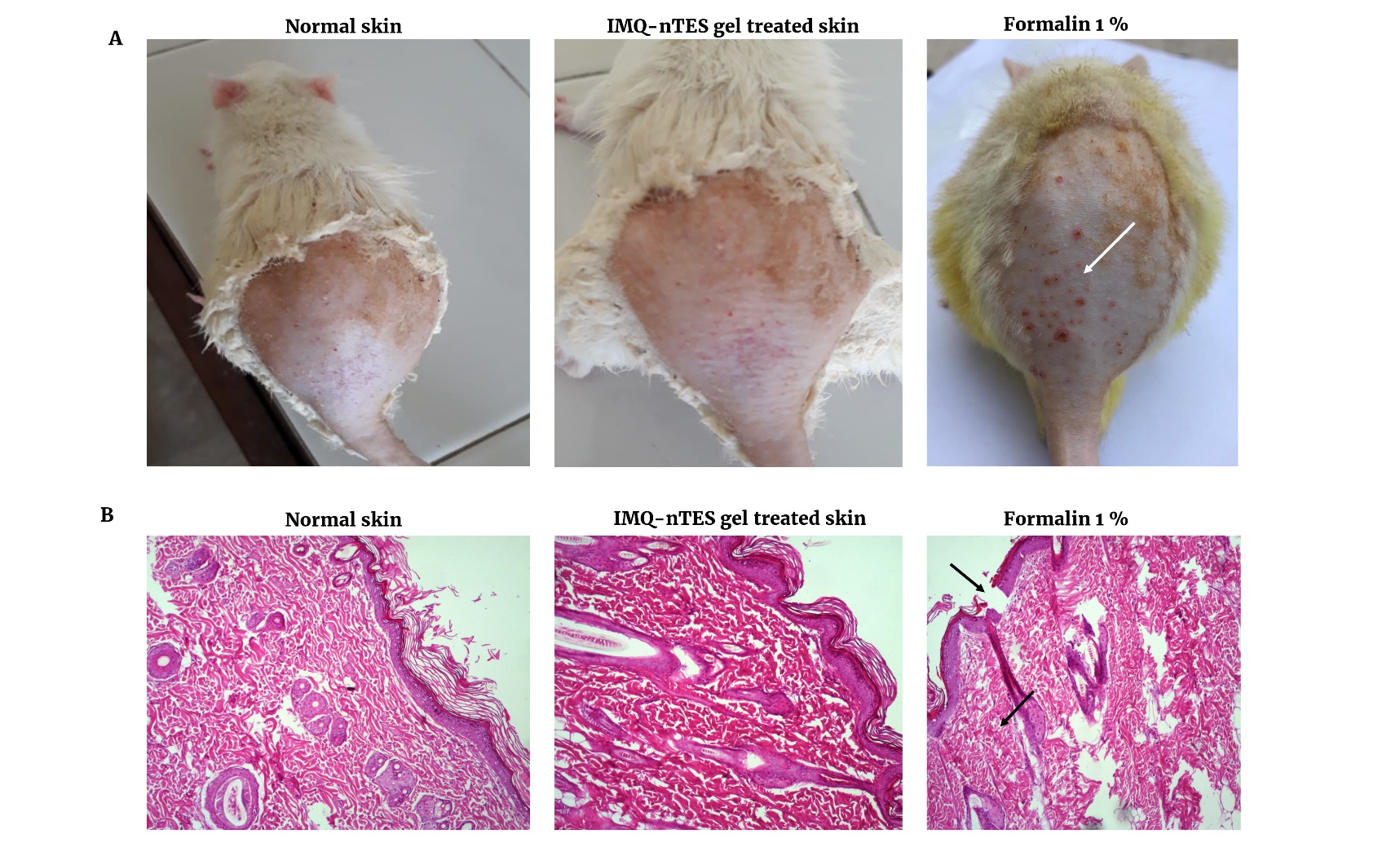


**Figure S2 Skin irritation study.**
